# Dual regulation of *lin28a* by Myc is necessary during zebrafish retina regeneration

**DOI:** 10.1083/jcb.201802113

**Published:** 2019-02-04

**Authors:** Soumitra Mitra, Poonam Sharma, Simran Kaur, Mohammad Anwar Khursheed, Shivangi Gupta, Mansi Chaudhary, Akshai J. Kurup, Rajesh Ramachandran

**Affiliations:** Indian Institute of Science Education and Research, Mohali, Knowledge City, Sector 81, SAS Nagar, Manauli, Mohali, Punjab, India

## Abstract

Cellular reprogramming leading to induction of Muller glia–derived progenitor cells (MGPCs) with stem cell characteristics is essential for zebrafish retina regeneration. Although several regeneration-specific genes are characterized, the significance of MGPC-associated Mycb induction remains unknown. Here, we show that early expression of Mycb induces expression of genes like *ascl1a*, a known activator of *lin28a* in MGPCs. Notably, *mycb* is simultaneously activated by Ascl1a and repressed by Insm1a in regenerating retina. Here, we unravel a dual role of Mycb in *lin28a* expression, both as an activator through Ascl1a in MGPCs and a repressor in combination with Hdac1 in neighboring cells. Myc inhibition reduces the number of MGPCs and abolishes normal regeneration. Myc in collaboration with Hdac1 inhibits *her4.1*, an effector of Delta–Notch signaling. Further, we also show the repressive role of Delta–Notch signaling on *lin28a* expression in post-injured retina. Our studies reveal mechanistic understanding of Myc pathway during zebrafish retina regeneration, which could pave way for therapeutic intervention during mammalian retina regeneration.

## Abstract (precis)

In zebrafish, the damaged retina can regenerate with the help of Muller glia–derived progenitor cells. Mitra et al. show that Mycb regulates *lin28a*, a facilitator of regeneration, both as an activator and repressor in selected cells. Further, Mycb in collaboration with Hdac1 represses *her4.1*, a negative regulator of retina regeneration.

## Graphical Abstract

**Figure:**
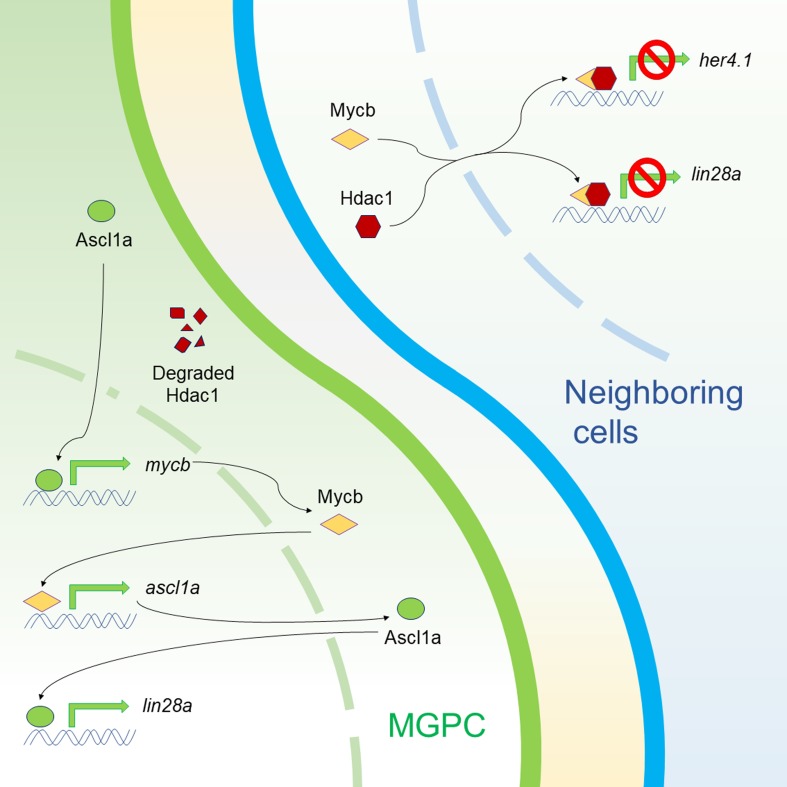

## Introduction

Compared with mammals, vertebrates such as fishes and amphibians have robust regenerative potential, which has facilitated better understanding of molecular mechanisms during tissue regeneration (Gemberling et al., 2013; Goldman, 2014; Mokalled et al., 2016; Ail and Perron, 2017; Rabinowitz et al., 2017). The zebrafish is extensively used to study regeneration of complex tissues such as retinae. Unlike mammals, zebrafish Muller glia (MG) possess remarkable ability to reprogram themselves to produce MG-derived progenitor cells (MGPCs), irrespective of the injury paradigms (Powell et al., 2016), which are capable of regenerating the damaged retina (Fausett and Goldman, 2006; Ramachandran et al., 2010b). Zebrafish retina regeneration is possible through the orchestration of various growth factors (Russell, 2003; Wan et al., 2012; Zhao et al., 2014b; Gramage et al., 2015), cytokines (Wan et al., 2014; Zhao et al., 2014b), gene transcription factors (Ramachandran et al., 2010a, 2012; Thummel et al., 2010; Nelson et al., 2012; Wan et al., 2014), epigenome modifiers (Powell et al., 2012, 2013; Mitra et al., 2018), cell cycle regulators (Ramachandran et al., 2011, 2012; Luo et al., 2012), Sonic hedgehog signaling–induced gene regulatory network (Kaur et al., 2018; Thomas et al., 2018), and differentiation factors (Munderloh et al., 2009) that are induced at the site of injury. Interestingly, mammalian MG exhibiting stem cell characteristics have been identified, which can be coaxed to grow and differentiate into retinal neurons to a limited extent (Ooto et al., 2004; Pollak et al., 2013; Ueki et al., 2015; Jorstad et al., 2017; Elsaeidi et al., 2018). Unraveling the complete cascade of gene regulatory network after zebrafish retina injury could help in deciphering the lack of efficient regeneration in mammals.

With the increasing knowledge of pluripotency-inducing factors (PIFs) in cellular reprogramming (Yu et al., 2007; Maekawa et al., 2011), studies have been undertaken to unravel the roles of naturally induced PIFs during MG reprogramming, leading to MGPC induction and retina regeneration (Ramachandran et al., 2010a; Reyes-Aguirre and Lamas, 2016; Yao et al., 2016; Gorsuch et al., 2017). However, the roles of an important PIF, Myc, during retina regeneration largely remain unknown. The c-Myc has been well characterized because of its impact on diverse biological functions. These include cellular transformation, cell cycle progression, escaping of the cell cycle arrest, inhibiting cell differentiation, and apoptosis (Amati and Land, 1994; Packham and Cleveland, 1995; Packham et al., 1996; Hoffman and Liebermann, 1998). The involvement of c-Myc in wound healing (Shi et al., 2015) and also after epithelial injury (Volckaert et al., 2013) is well documented. However, the roles of c-Myc with regards to regeneration are restricted to liver tissue of mice (Sobczak et al., 1989; Morello et al., 1990; Sanders et al., 2012) and rats (Arora et al., 2000), rat pancreas (Calvo et al., 1991), and *Xenopus laevis* limb (Lemaître et al., 1992) with limited knowledge about its actual mechanistic involvement.

The zebrafish has two Myc genes, namely *myca* and *mycb*. Here, we report the roles played by transcription factor Mycb in collaboration with Max, along with Ascl1a and Histone deacetylase1 (Hdac1), to regulate *lin28a* expression during MG reprogramming and induction of MGPCs. We show both the inductive and repressive roles played by Myc, enabling fine-tuned *lin28a* gene expression at the site of injury. Also, we mechanistically show the Mycb-influenced regulation of *hairy enhancer of split-related 4.1* (*her4.1*) during injury-dependent MG reprogramming, leading to MGPC induction and differentiation that culminate in regeneration.

## Results

### Myc expression is associated with MGPCs in post-injured retina

**Figure 1.**
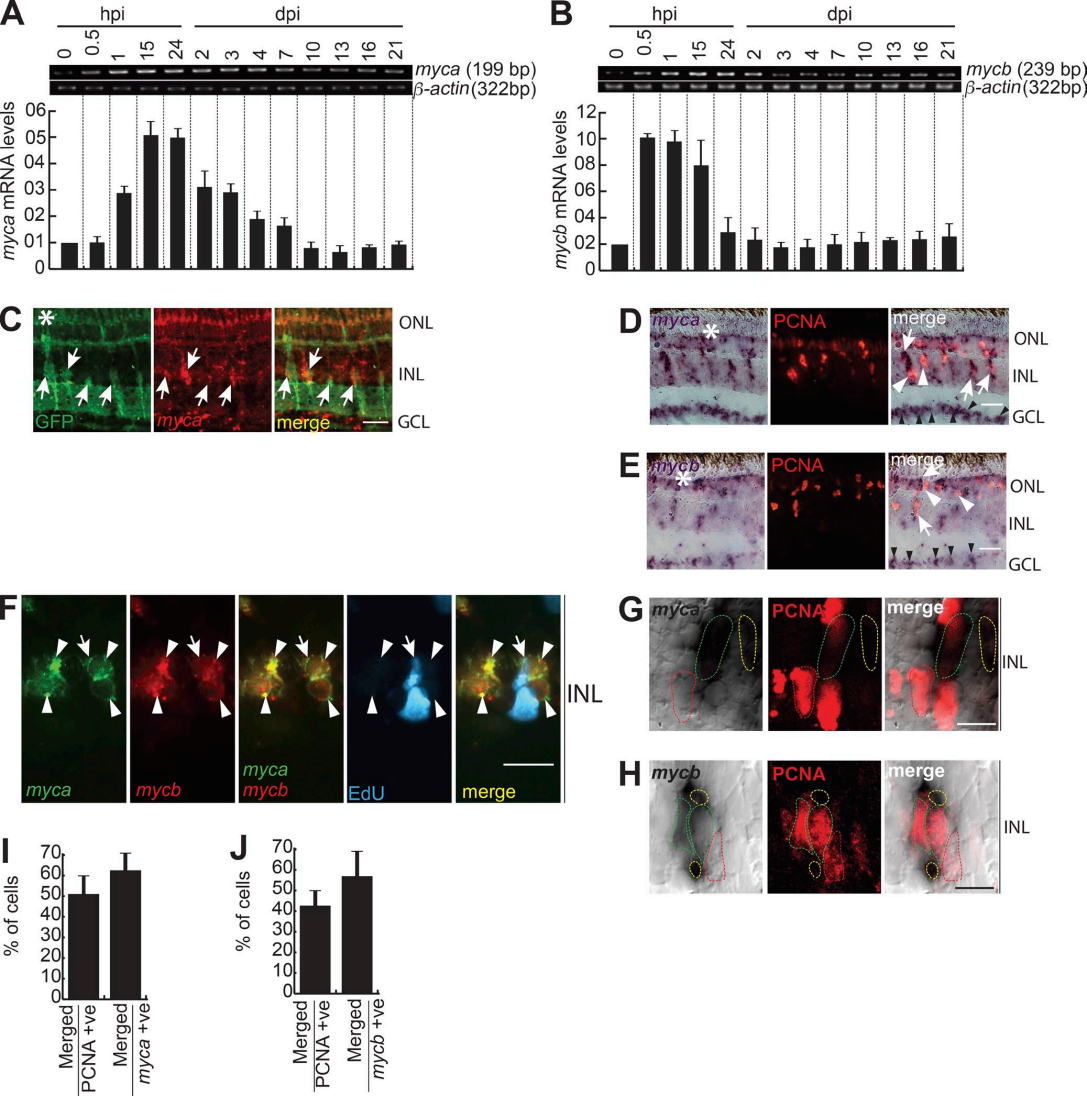
**Myc genes are rapidly induced in the injured retina. (A and B)** RT-PCR (top) and qPCR (bottom) were used to assay injury-dependent *myca* (A), and *mycb* (B) gene expressions; *n* = 6 biological replicates. **(C)** FISH and IF microscopy show expression of *myca* mRNA and GFP in retina of *1016 tuba1a:gfp* transgenic fish at 4 dpi. **(D and E)** ISH and IF microscopy show that *myca* (D) and *mycb* (E) mRNA is expressed in PCNA^+^ MGPCs and neighboring cells at 4 dpi. The white arrows indicate colabeled cells in D and E, and white arrowheads in D and E identify *myca*^−^ and *mycb*^−^ but PCNA^+^ cells near injury site. Black arrowheads indicate GCL-specific *myca* and *mycb* expression. **(F)** FISH microscopy shows coexpression of *myca* and *mycb* mRNA in BrdU^+^ cells and vicinity. The white arrow indicates EdU^+^, *myca*^+^, and *mycb*^+^ cells, and arrowheads mark *myca*^+^ and *mycb*^+^ cells. **(G–J)** A single 0.5-µm-thick Z section shows *myca* (G) and *mycb* (H) in 4-dpi retina; dotted outline in red shows PCNA^+^ *myca*^−^/*mycb*^−^ cells, green shows colabel with PCNA and *myca*/*mycb*, and yellow indicates *myca^+^*/*mycb^+^* but PCNA^−^ cells; and the percentage colabeling with PCNA is quantified (I and J); *n* = 5 biological replicates. Bars, 10 µm; white asterisks mark the injury sites. ONL, outer nuclear layer; INL, inner nuclear layer (C–H).

The Myca and Mycb isoforms show 80% amino acid identity (Fig. S1 A). The *mycb* expression was seen as early as 2 h of embryonic development, indicating its importance (Fig. S1 B). When their mRNA levels were examined after retinal injury by quantitative PCR (qPCR) and reverse transcription PCR (RT-PCR; Fig. 1, A and B), *mycb* showed an early expression-peak compared with *myca.* The mRNA in situ hybridization (ISH) of both *myca* and *mycb* exhibited a panretinal expression pattern at 12 h post injury (hpi) that became restricted to the injury site by 2 d post injury (dpi; Fig. S1, C and D). The *myca* expression was seen in both GFP^+^ and adjacent cells of *1016 tuba1a:gfp* transgenic fish retina, in which MGPCs are marked with GFP upon injury (Fig. 1 C and Fig. S1 E; Fausett and Goldman, 2006). Both *myca* and *mycb* were expressed in proliferating cell nuclear antigen (PCNA)^+^/EdU^+^ MGPCs and adjacent cells at 4–6 dpi (Fig. 1, D–F; and Fig. S1, C and D). We also found specific up-regulation of *myca* and *mycb* in ganglion cell layer (GCL; Fig. 1, D and E), suggestive of their roles in optic nerve regeneration as well. In support of this, we found a strong ganglion layer–specific expression of *mycb* upon optic nerve lesion (Fig. S1 F). Notably, a closer evaluation of 4-dpi retina revealed that both *myca* and *mycb* are often associated with cells flanking PCNA^+^ MGPCs (Fig. 1, G and H). Approximately 40% of the PCNA^+^ cells expressed *myca* and *mycb*, whereas 60% of the *myca*^+^ and *mycb^+^* cells had PCNA (Fig. 1, I and J). Spatial and temporal expression pattern of *myca* and *mycb* was seemingly reminiscent to previously reported genes like *ascl1a*, *insm1a*, and *hb-egf* (Ramachandran et al., 2010a, 2012; Wan et al., 2012). These observations suggest the existence of a *myca*/*mycb*-mediated reprogramming to induce MGPCs in damaged retina.

### Myc expression and activity in post-injured retina is essential for regeneration

**Figure 2.**
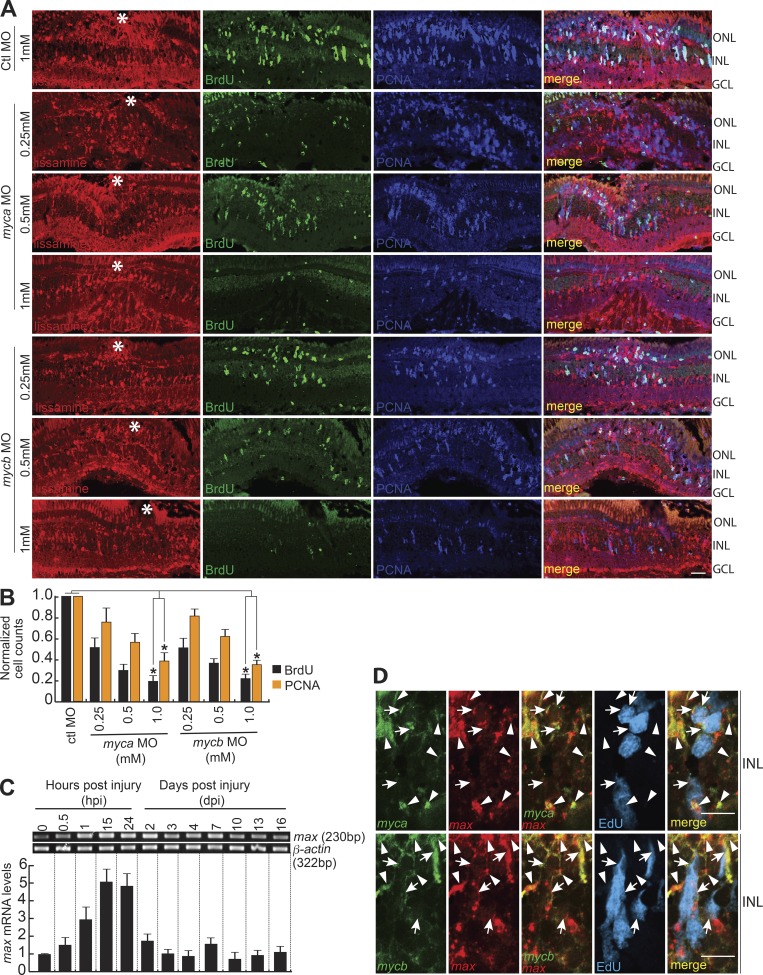
**Myc is necessary for MG dedifferentiation in the injured retina. (A)** IF microscopy images of control (Ctl; 1 mM concentration) or *myca*/*mycb*-targeting lissamine-labeled MOs (0.25, 0.5, and 1 mM concentration each), electroporated into the retina of zebrafish at the time of retinal injury shows a concentration-dependent decrease in the number of MGPCs. Fish were given an intraperitoneal injection of BrdU, 3 h before euthanasia on 4 dpi. The white asterisks mark the injury sites. **(B)** Quantification of the number of BrdU^+^ and PCNA^+^ cells at the injury site. The data are compared with control MO. *, P < 0.001; *n* = 4 biological replicates. **(C)** RT-PCR (top) and qPCR (bottom) were used to assay injury-dependent *max* gene expression; *n* = 6 biological replicates. **(D)** ISH and IF microscopy show that *max* gene expression colabel with *myca* and *mycb* mRNA in EdU^+^ MGPCs and other surrounding cells at 4 dpi. White arrowheads indicate *myca* or *mycb* colabeled with *max*, and white arrows mark myca/mycb/max in EdU^+^ MGPCs. Bars, 10 µm (A and D). Error bars are SD. ONL, outer nuclear layer; INL, inner nuclear layer.

Lissamine-tagged, morpholino-modified antisense oligonucleotides (MOs) targeting *myca* and *mycb* completely blocked the translation of respective mRNA in retina (Fig. S1 G), and GFP mRNA appended with MO-binding sites, injected in embryos (Fig. S1 H). Importantly, *myca*/*mycb* knockdown using two MOs that target different regions of their mRNAs, electroporated into freshly injured retina, showed that Myca and Mycb are necessary for the generation of BrdU^+^/PCNA^+^ MGPCs (Fig. 2, A and B; and Fig. S1, I and J). It is interesting to note that *myca* and *mycb* double knockdown had an additive effect on the total number of BrdU^+^ cells (Fig. S2, A and B). These observations indicate the possibility of independent pathways mediated by Myca and Mycb that converge to induce MGPCs in injured retina. Notably, the decline in the proliferating population of MGPCs in *myca*/*mycb* MO-electroporated retina was not because of increased rate of cellular apoptosis revealed in a TUNEL assay (Fig. S2, C and D). Furthermore, the transfection of *myca*/*mycb* and *gfp* reporter mRNAs, along with respective MOs that block endogenous, but not the delivered mRNAs, could rescue the reduction in cell proliferation in the retina at 4 dpi (Fig. S3, A–C).

**Figure 3.**
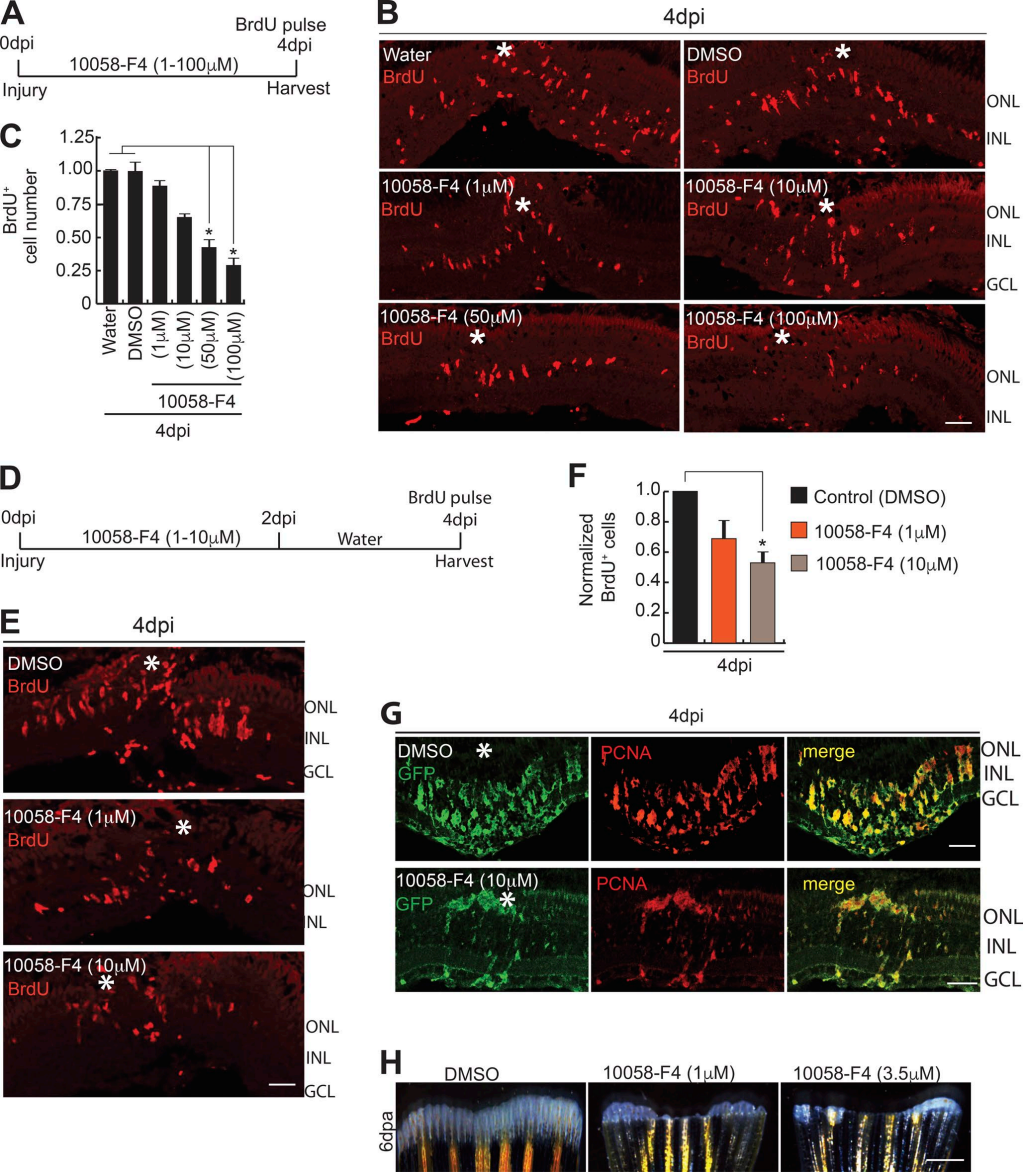
**Blockade of Myc–Max interaction abolishes MGPC proliferation in retina and fin regeneration. (A–C)** Blockade of the Myc–Max interaction using the drug 10058-F4 treatment, as shown in timeline of experiment (A), reveals significant reduction in BrdU^+^ MGPCs at 4 dpi, seen by IF microscopy (B), which is quantified and normalized to Water/DMSO control (C). *, P < 0.0001; *n* = 5 biological replicates. **(D–F)** Early treatment with 10058-F4, as shown in timeline of experiment (D), also reveals a significant reduction in BrdU^+^ MGPCs at 4 dpi revealed by IF microscopy (E), which is quantified and normalized to DMSO control (F). *, P < 0.01; *n* = 5 biological replicates. **(G)** IF microscopy analysis of GFP and PCNA after 10058-F4 treatment shows a reduction in the number of MGPCs in *1016 tuba1a:gfp* transgenic fish retina at 4 dpi. **(H)** Regenerating fin-blastema shows a decline in cell mass in 10058-F4–treated post-amputated fin (1 µM and 3.5 µM) at 6 d after amputation, compared with DMSO control. Error bars are SD. Bars: 10 µm (B, E, and G) and 500 µm (H). White asterisks mark the injury sites (B, E, and G). ONL, outer nuclear layer; INL, inner nuclear layer.

One of the basic helix-loop-helix leucine zipper family members, Max, known to interact with Myc (Yin et al., 2003; Ecevit et al., 2010), showed similar expression pattern as *myca* and *mycb* in post-injured retina (Fig. 2, C and D; and Fig. S2 E) and optic nerve lesion (Fig. S3, D–F). Max is also an obligatory partner for Myc’s gene transactivation functions (Amati and Land, 1994). Furthermore, *max* coexpressed with *mycb* in GFP^+^ MGPCs of *1016 tuba1a:gfp* transgenic retina at 4 dpi (Fig. S3 G). The cell-sorting analysis from *1016 tuba1a:gfp* transgenic retina revealed an increased expression levels of *myca*, *mycb*, and *max* in GFP^+^ MGPCs, in comparison to rest of the GFP^−^ retinal cell types at 4 dpi (Fig. S3, H and I). These results from zebrafish retina and other reports (Yin et al., 2003; Wang et al., 2007) emphasize that Myc functions mainly in combination with Max to activate transcription and stimulate cell proliferation. This made us explore the effects of disruption of Myc–Max interaction during retina regeneration, using a pharmacological inhibitor, 10058-F4, which blocks Myc–Max interaction (Yin et al., 2003; Huang et al., 2006; Lin et al., 2007; Wang et al., 2007). This would also be an alternate way of inhibiting Myc function. We found that 10058-F4 treatments blocked up to 70% of cell proliferation in regenerating WT retina with continuous (Fig. 3, A–C) or discontinuous exposure (Fig. 3, D–F). Furthermore, we also found a drastic decline in PCNA^+^/GFP^+^ MGPCs in *1016 tuba1a:gfp* retina (Fig. 3 G) at 4 dpi, according to an experimental time line (Fig. 3 A). However, there was no significant change in the rate of apoptosis in 10058-F4–treated retina, as revealed in TUNEL assay (Fig. S3, J and K). There was also no visible change in expression levels of various genes in retina at 4 dpi because of the DMSO present as solvent in the drug 10058-F4 (Fig. S3 L). These results suggest the importance of Myc–Max interaction in dedifferentiation and proliferation phases of retina regeneration. Notably, the inhibition of Myc also negatively affected fin regeneration (Fig. 3 H), suggesting that normal Myc–Max interaction may be necessary during regeneration of different tissues.

### Myc–Ascl1a cross-talk during regeneration

**Figure 4.**
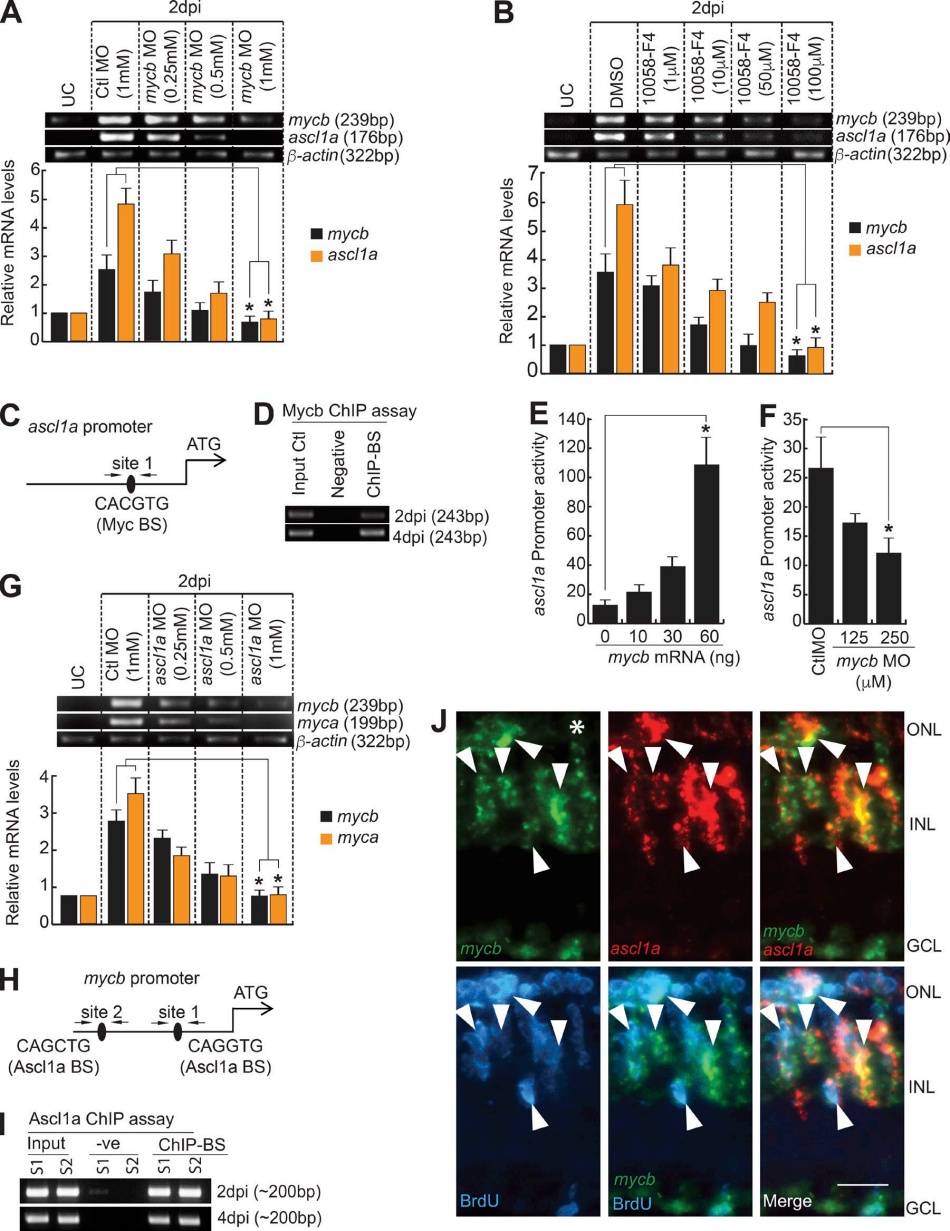
**Myc-Ascl1a coexpression and interdependency during regeneration. (A and B)** RT-PCR (top) and qPCR (bottom) show inhibition of *mycb* through MO (A), and 10058-F4 (B) down-regulates *ascl1a* and *mycb* induction, relative to the control at 2 dpi. *, P < 0.0001 in A and B; *n* = 6 biological replicates. **(C and H)** Diagram of *ascl1a* (C) and *mycb* (H) promoters with putative Mycb (C)- and Ascl1a (H)-binding sites. The solid lines represent DNA sequences of the promoter. Arrows mark ChIP primers, and capital letters mark consensus sequence. **(D and I)** The retina ChIP assay done at 2 and 4 dpi shows that Myc bound to *ascl1a* promoter (D), and Ascl1a bound to *mycb* promoter (I). **(E)** Mycb overexpression up-regulates *ascl1a:gfp-luciferase* expression in embryos. *, P < 0.01. **(F)** MO-based Myc blockade inhibits *ascl1a:gfp-luciferase* in embryos. Promoter activity is normalized light units with internal control *Renilla* luciferase. *, P < 0.001. **(G)** RT-PCR (top) and qPCR (bottom) show MO based *ascl1a* knockdown down-regulates *myca* and *mycb* expression in 2 dpi retina. *, P < 0.002. **(J)** FISH and IF microscopy show colocalization of *mycb* and *ascl1a* expressing cells with BrdU^+^ MGPCs in 4-dpi retina. Arrowheads indicate colabeled *mycb*- and *ascl1a*-expressing cells. White asterisk marks the injury site. Bars, 10 µm. *n* = 6 biological replicates unless specified. Error bars are SD. S, site; −ve, negative, BS, binding site.

We found that *myca* and *mycb* were induced immediately after retinal injury, which is temporally ahead of previously reported *ascl1a* induction regimen (Ramachandran et al., 2010a). We speculated a hierarchical relationship between them. Mycb inhibition using antisense MO or the drug 10058-F4 down-regulated *ascl1a* and *mycb* (Fig. 4, A and B) and up-regulated *myca* and *max* (Fig. S4, A and B). Unlike several Myc-regulated genes (Dang, 1999; Fernandez et al., 2003; Zeller et al., 2003; Reymann and Borlak, 2008), which get affected by 10058-F4 treatment, other Myc family genes such as *mycn* and *mycla*, which showed a temporal variation in expression after retinal injury (Fig. S4 C), did not show a significant change (Fig. S4 D). Examination of *ascl1a* promoter revealed one putative Myc-binding site (Fig. 4 C) and chromatin immunoprecipitation (ChIP) assay using anti-Myc antibody on 2- and 4-dpi retina showed that Myc binds to this predicted site (Fig. 4 D). Zebrafish embryos coinjected with *ascl1a:gfp-luciferase* reporter and increasing concentrations of *mycb* mRNA and *mycb* MO separately, showed that Mycb stimulates *ascl1a* promoter activity (Fig. 4, E and F). This finding also suggests a developmental conservation in the regulation of these genes similar to that found in retina.

Knockdown of *ascl1a* resulted in a small but significant decrease in *myca* and *mycb* expression at 2 dpi (Fig. 4 G), which is similar to that seen with blockade of Wnt signaling through the drug XAV939 (Fig. S4, E and F). Blockade of Wnt signaling in regenerating retina is known to down-regulate *ascl1a* (Ramachandran et al., 2011). From these observations, we speculated the existence of direct and mutual regulatory relationship between Mycb and Ascl1a. Examination of *mycb* promoter sequence revealed two putative Ascl1a-binding sites (Fig. 4 H) and ChIP assay on 2- and 4-dpi retina confirmed that endogenous Ascl1a indeed bound onto these sites (Fig. 4 I), which is also supported by colocalization of *mycb* and *ascl1a* mRNAs (Fig. 4 J). These results strongly support the view that Ascl1a and Mycb up-regulate expression of each other during retina regeneration.

### Restricted expression of *mycb* is through transcriptional repressor Insm1a

**Figure 5.**
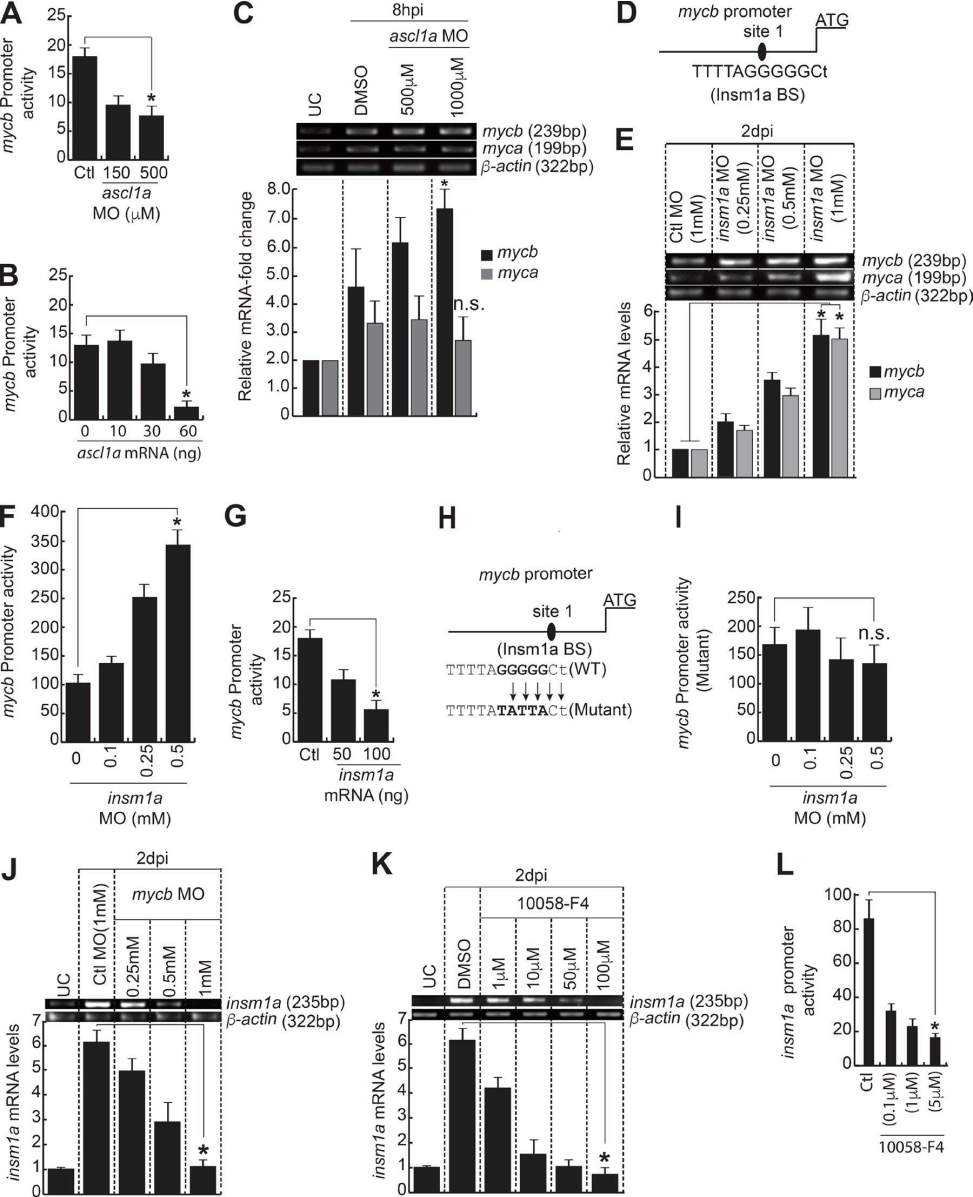
**Insm1a inhibits *mycb* expression in regenerating retina. (A)** MO-based gene knockdown of *ascl1a* down-regulates *mycb:gfp-luciferase* expression in embryos. *, P < 0.002. **(B)** Ascl1a overexpression inhibits *mycb:gfp-luciferase* expression in embryos. *, P < 0.0002. **(C)** MO-mediated *ascl1a* inhibition in retina as early as 8 h after injury causes an increase in *mycb*, but not *myca* expression. *, P < 0.009; *n* = 3 biological replicates. n.s., not significant. **(D)** Diagram of *mycb* promoter with putative Insm1a binding site. **(E)** MO-based *insm1a* knockdown significantly up-regulated both *myca* and *mycb* expression in injured retina at 2 dpi. *, P < 0.001. **(F)** The *insm1a* knockdown through MO up-regulates *mycb:gfp-luciferase* expression in zebrafish embryos by luciferase assay. *, P < 0.0001. **(G)** Insm1a overexpression inhibits *mycb:gfp-luciferase* expression in embryos. *, P < 0.001. **(H)** Schematic of *mycb* promoter with mutated Insm1a-binding site. **(I)** Insm1a inhibition through MO has no effect on mutated *mycb:gfp-luciferase* expression in zebrafish embryos by luciferase assay. n.s., not significant. **(J and K)** Myc inhibition through MO (J) and 10058-F4 (K) cause significant down-regulation of *insm1a* expression in 2-dpi retina. **(L)** 10058-F4–based Myc blockade inhibits *insm1a:gfp-luciferase* in embryos. Promoter activity is normalized light units with internal control *Renilla* luciferase. *, P < 0.0002. Error bars are SD. BS, binding site.

The *ascl1a* knockdown in zebrafish embryos coinjected with *mycb:gfp-luciferase* reporter with increasing concentrations of *ascl1a* MO showed a dose-dependent reduction in *mycb* promoter activity (Fig. 5 A), in agreement with retinal data, as discussed earlier (Fig. 4, G–J). However, overexpression of Ascl1a in zebrafish embryos coinjected with *mycb:gfp-luciferase* reporter, along with increasing concentrations of *ascl1a* mRNA, showed a surprising reduction in *mycb* promoter activity (Fig. 5 B). Interestingly, we found an increase in the *mycb* expression in the retina with *ascl1a* knockdown at an early time point of 8 hpi (Fig. 5 C), which suggests the involvement of some other transcriptional repressor molecule, regulated through Ascl1a, that affects *mycb* expression. We speculated that such a conundrum could be because of involvement of a potential intermediate repressor like Insm1a, which is induced by Ascl1a (Ramachandran et al., 2012).

We decided to explore if Insm1a-mediated gene repression is the possible cause of reduced *mycb* promoter activity in zebrafish embryos with Ascl1a overexpression and increased *mycb* mRNA levels in 8-hpi retina with *ascl1a* knockdown (Fig. 5, B and C). Examination of *mycb* promoter revealed one putative Insm1a-binding site (Fig. 5 D). The early panretinal and the late MGPC-associated expression of *insm1a* are important in stringent control of several regeneration-associated genes and cell cycle exit during retina regeneration (Ramachandran et al., 2012). To evaluate this further, we examined the expression levels of *myca* and *mycb* in *insm1a* knockdown background in injured retina. We found a dose-dependent increase in *myca* and *mycb* mRNA levels in retina upon MO-mediated *insm1a* knockdown, which supported its possible inhibitory role (Fig. 5 E). We evaluated this further through coinjection of *mycb:gfp-luciferase* reporter in zebrafish embryos, along with *insm1a* MO or *insm1a* mRNA separately. The results showed expected up-regulation and down-regulation in *mycb* promoter activity, respectively (Fig. 5, F and G). Furthermore, the mutation of the single Insm1a-binding site in *mycb* promoter abolished the increase in promoter activity when coinjected with *mycb:gfp-luciferase* reporter and different concentrations of *insm1a* MO in zebrafish embryos (Fig. 5, H and I). These results suggest that Ascl1a-mediated induction of Insm1a could be the cause of reduction of *mycb* promoter activity, seen by coinjection of zebrafish embryos with *mycb:gfp-luciferase* reporter and increasing concentrations of *ascl1a* mRNA.

Similarly, the *ascl1a* knockdown-mediated increase in *mycb* mRNA seen in 8-hpi retina (Fig. 5 C) also could be because of decline in Insm1a. Earlier studies showed that induction of *ascl1a* is panretinal in nature, wherein both MG and non-MG cells of the retina express it at 6–8 hpi (Ramachandran et al., 2011, 2012). This kind of panretinal induction of Ascl1a, soon after injury, up-regulates the transcriptional repressor Insm1a throughout the retina. Insm1a is also necessary for repressing *ascl1a* and its own expression at early stages of regeneration. The Ascl1a–Insm1a regulatory loop is a prelude for initiation of MG dedifferentiation (Ramachandran et al., 2012). However, at 4 dpi, approximately only 40% of the MGPCs, which were exiting the cell cycle, expressed *insm1a* (Ramachandran et al., 2012). Such a stringent gene regulation in the retina would be essential for restricting the *mycb* expression to the vicinity of MGPCs at the site of injury from its initial panretinal induction. Furthermore, *mycb* knockdown and 10058-F4 treatments separately caused down-regulation of *insm1a* in 2-dpi retina (Fig. 5, J and K). Moreover, a decrease in *insm1a* promoter activity was seen in zebrafish embryos injected with *insm1a:gfp-luciferase* reporter and exposed to various concentrations of 10058-F4 (Fig. 5 L). These observations could be the result of decline in Ascl1a, which is an inducer of *insm1a* (Ramachandran et al., 2012). Collectively, these experiments reveal an efficient Myc–Ascl1a–Insm1a regulatory loop in action that contributes to the course of regeneration.

### Mycb-mediated dual regulation of *lin28a* during regeneration

**Figure 6.**
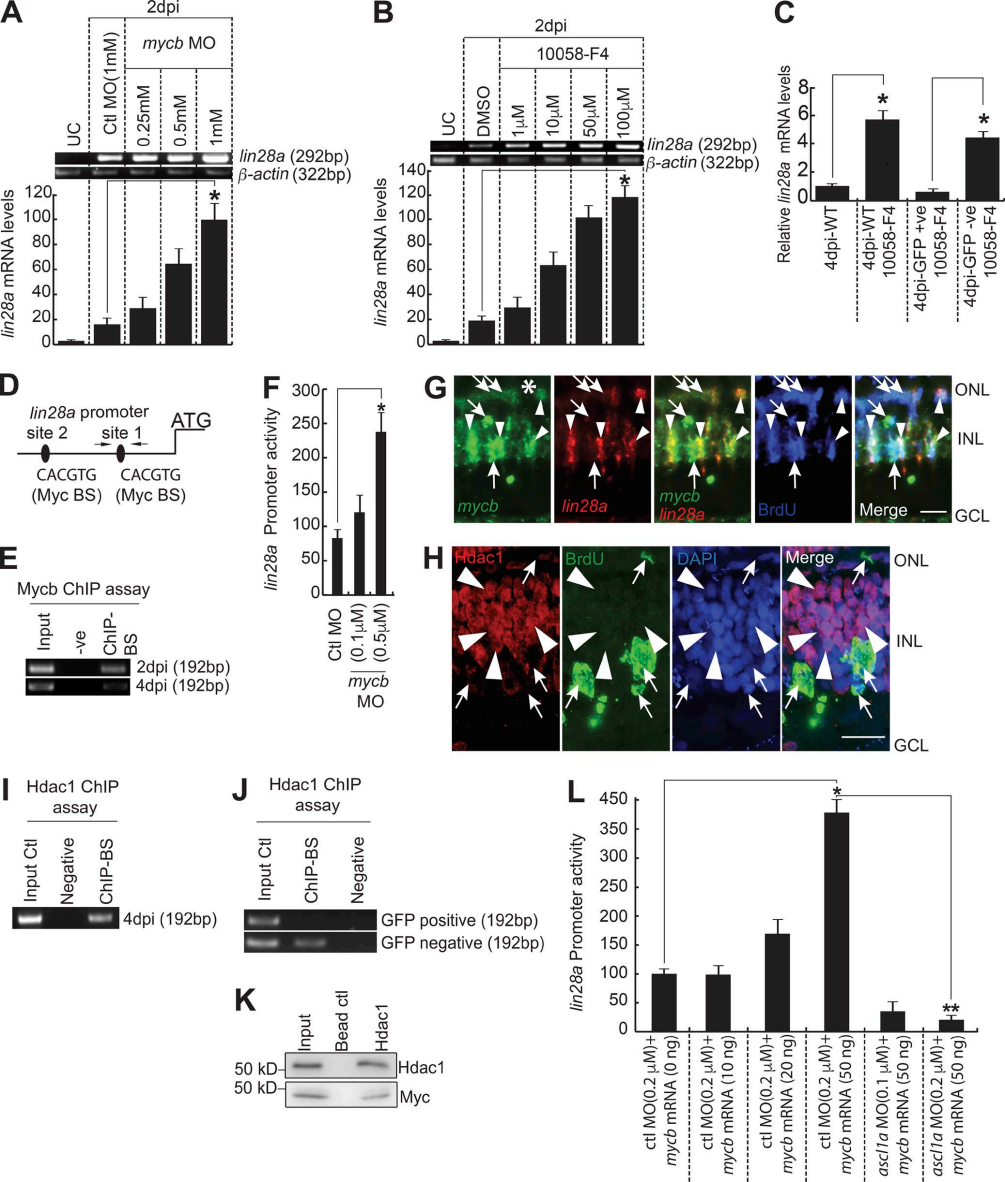
**Mycb-mediated regulation of *lin28a* in MGPCs. (A and B)** RT-PCR (top) and qPCR (bottom) show Myc inhibition using antisense MO (A) or 10058-F4 (B) induce *lin28a* in 2-dpi retina. *, P < 0.001. UC, uninjured control. **(C)** qPCR analysis of *lin28a* mRNA from GFP^+^ and GFP^−^ cells sorted from *1016 tuba1a:gfp* transgenic fish retina with 1-µM 10058-F4 treatment at 4 dpi, compared with WT. *, P < 0.01. **(D)** Diagram of *lin28a* promoter with putative Mycb-binding sites. The solid lines represent DNA sequences. Arrows mark ChIP primers, and capital letters mark consensus sequence. **(E)** The retina ChIP assay at 2 and 4 dpi showed that Myc binds to *lin28a* promoter. **(F)** MO-based *mycb* knockdown up-regulates *lin28a:gfp-luciferase* activity. Promoter activity is normalized light units with internal control *Renilla* luciferase. *, P < 0.0002. **(G)** FISH and IF microscopy show expression of *mycb* and *lin28a* with respect to BrdU^+^ MGPCs in 4-dpi retina. Arrowheads indicate colabel of *mycb*, *lin28a*, and BrdU^+^ cells; arrows indicate *mycb*^+^ that colabel with BrdU, but are *lin28a*^−^ cells. White asterisk marks the injury site. **(H)** IF and ISH microscopy on a single 0.5-µm thick Z section shows Hdac1 expression secludes largely from BrdU^+^ cells at the site of injury. Arrowheads mark *hdac1*^+^ but BrdU^−^ cells, and arrows mark Hdac1^−^ but BrdU^+^ cells. **(I)** ChIP assay using Hdac1 antibody reveals Hdac1 occupied Myc-binding site on *lin28a* promoter. **(J)** ChIP assay of *lin28a* promoter Myc-binding region, using Hdac1 antibody from GFP^+^ and GFP^−^ cells from *1016 tuba1a:gfp* transgenic retina. **(K)** Co-IP assay using Hdac1 antibody reveals Hdac1–Myc collaboration during retina regeneration. **(L)** MO-based *ascl1a* knockdown abrogates Mycb overexpression-mediated *lin28a:gfp-luciferase* up-regulation in embryos. *, P < 0.0002; **, P < 0.003. *n* = 6 biological replicates unless specified. Error bars are SD. −ve, negative; +ve, positive; BS, binding site. Bars, 10 µm (G and H). ONL, outer nuclear layer; INL, inner nuclear layer.

Since we found the regulation of *ascl1a* and *insm1a* through Mycb, we then probed for the expression pattern of an important regeneration-associated gene, *lin28a*, both in *mycb* knockdown and in the absence of its activity. Interestingly, absence of Myc caused a significant up-regulation of *lin28a* in WT retina (Fig. 6, A and B; and Fig. S4, G and H). To assess if there is any cell type bias in up-regulation of *lin28a* with Myc inhibition, we used *1016 tuba1a:gfp* transgenic fish retina. We found a selective increase in the *lin28a* mRNA levels in GFP^−^ cells compared to the GFP^+^ mRNA levels of *1016 tuba1a:gfp* fish treated with 10058-F4 (Fig. 6 C). Examination of *lin28a* promoter revealed two putative Myc-binding sites (Fig. 6 D). ChIP assay done on 2- and 4-dpi retina confirmed endogenous Myc bound onto one of the two sites (Fig. 6 E). This was further confirmed by promoter activity assay in zebrafish embryos coinjected with *lin28a:gfp-luciferase* reporter and *mycb* MO (Fig. 6 F). Interestingly, *lin28a* mRNA showed a colocalization with only a subset of *mycb^+^* cells, but stayed secluded in some neighboring MGPCs (Fig. 6 G). These results made us explore the possible ways in which Mycb could act as a repressor of *lin28a*. Myc is known to recruit bona fide transcription repressors like histone deacetylases (Hdacs) to suppress target genes (Kurland and Tansey, 2008). We speculated that similar mechanism might regulate Myc-mediated down-regulation of *lin28a*. To support this view further, we also saw a decline in Hdac1 expression in proliferating MGPCs pulse-labeled with BrdU at 4 dpi (Fig. 6 H). Surprisingly, we found the occupation of Hdac1 at Myc-binding sites of *lin28a* promoter, as revealed in a ChIP assay done in whole retina, using the same set of primers used for confirming the Myc-binding site on *lin28a* promoter (Fig. 6 I). Again, we did not find Hdac1 binding in Mycb-recognized DNA sequence of *lin28a* promoter in GFP^+^ MGPCs isolated from *1016 tuba1a*:GFP transgenic retina (Fig. 6 J). Furthermore, to confirm the physical interaction of Myc and Hdac1, we performed a coimmunoprecipitation (co-IP) assay using Hdac1 antibody in 4-dpi retinal extract. We found that the protein complex, pulled down using Hdac1 antibody, contained Myc protein (Fig. 6 K), suggesting the existence of a physical collaboration between Hdac1 and Myc in causing repression of *lin28a*.

However, when zebrafish embryos were coinjected with *lin28a:gfp-luciferase* reporter and *mycb* mRNA, we observed a concentration-dependent up-regulation of *lin28a* promoter activity (Fig. 6 L). We speculated that Myc-mediated induction of Ascl1a, an activator of *lin28a*, could be the cause of increased *lin28a* promoter activity. If this is true, the effect of Ascl1a on *lin28a* promoter could be nullified by using *ascl1a* MO. We found a drastic decline in *lin28a* promoter activity in zebrafish embryos coinjected with a constant high *mycb* mRNA dose and increasing concentrations of *ascl1a* MO (Fig. 6 L), confirming our speculation. These results suggest that Mycb could impact the *lin28a* promoter indirectly as an activator in BrdU-positive MGPCs through Ascl1a and a repressor in combination with Hdac1 in BrdU-negative neighboring cells at the site of injury.

### Mycb regulates *her4.1* through Hdac1 in injured retina

Delta–Notch signaling–mediated regulation of cell proliferation during retina regeneration is well characterized in zebrafish. Inhibition of Delta–Notch signaling through administration of γ-secretase inhibitor *N*-(*N*-[3,5-difluorophenylacetyl]-l-alanyl)-*S*-phenylglycine-*t*-butyl ester (DAPT) caused an increase in MGPC proliferation in the injured retina (Wan et al., 2012; Conner et al., 2014). The DAPT treatment also caused an expected decline in expression levels of target genes of Delta–Notch signaling, such as *her4.1*, compared with control retina (Fig. S5 A). Furthermore, the induced expression of notch intracellular domain (*nicd*) in the retina caused panretinal expression of *her4.1* (Kageyama et al., 2007; Zhou et al., 2012). The overexpression of *nicd* is also associated with negligible MGPC proliferation and retina regeneration (Wan et al., 2012).

**Figure 7.**
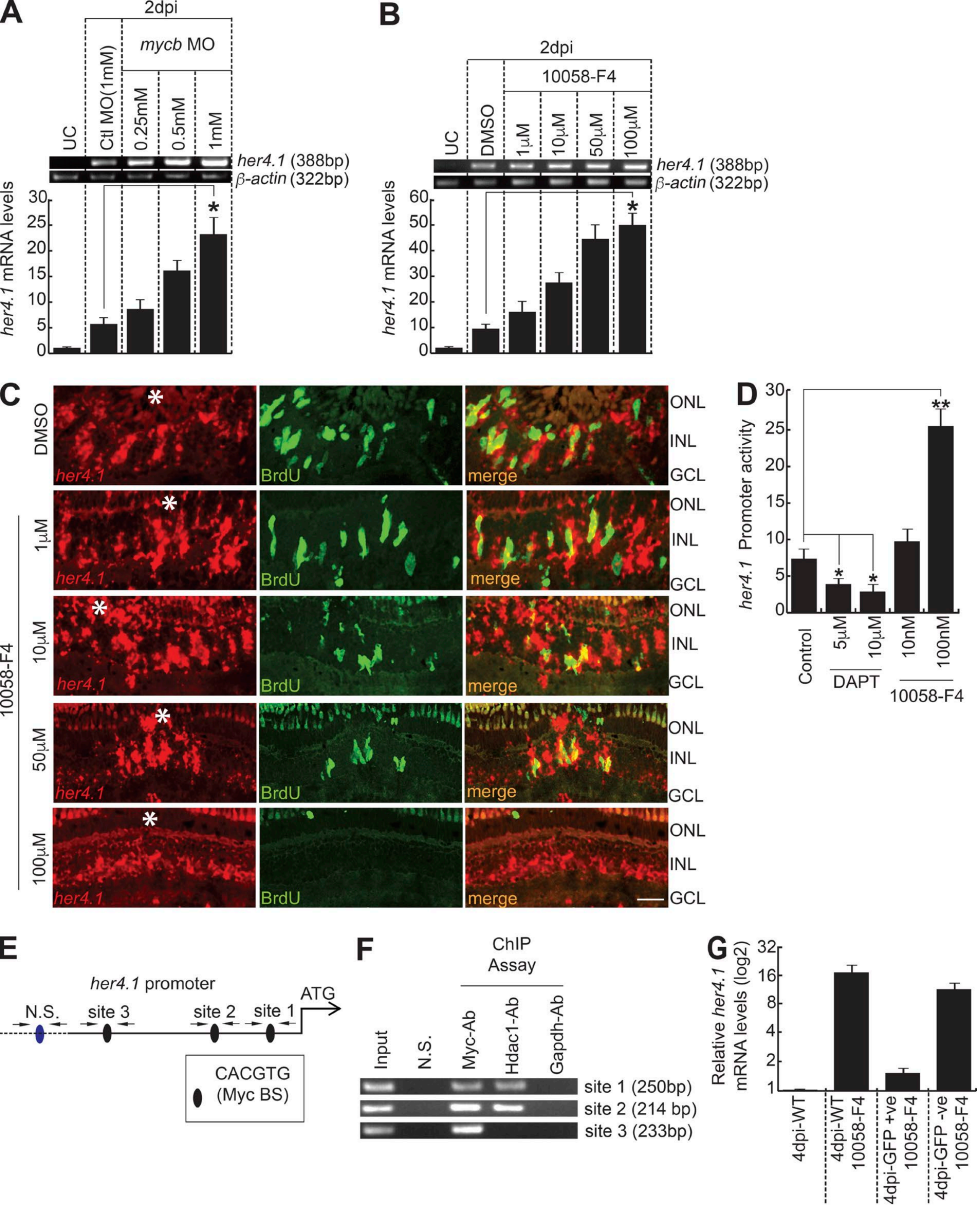
**Mycb regulates *her4.1* gene transcription in injured retina. (A and B)** RT-PCR (top) and qPCR (bottom) show increased *her4.1* induction with MO-based *mycb* knockdown (A) or 10058-F4 (B) relative to control MO and DMSO, respectively, in 2-dpi retina. *, P < 0.005. UC, uninjured control. **(C)** FISH and IF microscopy shows that 10058-F4 treatment increases *her4.1* expression compared with water or DMSO-treated control in 4-dpi retina. Bar, 10 µm. White asterisks mark the injury sites. **(D)** Myc inhibition through 10058-F4 up-regulates *her4.1:gfp-luciferase* expression compared with control and DAPT-treated embryos. **(E)** Diagram of *her4.1* promoter with putative Mycb-binding sites. The solid lines represent DNA sequences of the promoter. **(F)** The retina ChIP assay at 4 dpi reveals Myc and Hdac1 bound to Myc-BS on *her4.1* promoter. **(G)** qPCR analysis of *her4.1* mRNA from GFP^+^ and GFP^−^ MGPCs sorted from *1016 tuba1a:gfp* transgenic fish retina with 1 µM 10058-F4 treatment at 4 dpi, compared with WT. *n* = 3 biological replicates in all experiments. ONL, outer nuclear layer; INL, inner nuclear layer; N.S., nonspecific; BS, binding site.

Here, we found a significant decline in MGPC proliferation because of Myc inhibition, similar to that of *nicd* overexpression. Based on these observations, we speculated the existence of the mechanistic involvement of Her4.1, which could cause a compromised MGPC proliferation and regeneration in the Myc-inhibited retina. In support of this hypothesis, we found a significant up-regulation of *her4.1* in response to *mycb* knockdown and 10058-F4 treatments at 2 dpi revealed by RT-PCR and qPCR (Fig. 7, A and B). Compared with the normal injury-restricted expression of *her4.1* at 4 dpi, its panretinal induction was seen with Myc inhibition, by 10058-F4 treatments in a dose-dependent manner (Fig. 7 C and Fig. S5 B). We also found similar induction of *her4.1* because of indirect inhibition of *mycb* through XAV939-mediated blockade of Wnt signaling (Fig. S4 E and Fig. S5 C). Finally, 10058-F4–treated zebrafish embryos coinjected with *her4.1:gfp-luciferase* reporter caused an increase in *her4.1* promoter activity, while DAPT caused the opposite (Fig. 7 D). These results suggested that the involvement of Myc–Her4.1 interplay is necessary to restrict the MGPC proliferation to the site of injury during retina regeneration.

We explored further to find if Myc directly regulates *her4.1* through direct interactions onto its promoter sequences. A similar case was reported, wherein another member of Notch target genes, *hes1*, is up-regulated through sonic hedgehog signaling–dependent direct target Gli2 in retinal progenitors, which is independent of classical Delta–Notch signaling (Wall et al., 2009). In silico analysis of *her4.1* regulatory sequences revealed a few putative Myc-binding sites (Fig. 7 E). Since we already demonstrated physical interaction of Mycb and Hdac1 and its occupation in *lin28a* regulatory sequences to cause its repression in regenerating retina (Fig. 6, I–K), we speculated that such a scenario could underlie the regulation of *her4.1* as well. We performed a ChIP assay in 4-dpi retinal chromatin using antibodies against Myca/b, Hdac1, and Gapdh. Interestingly, we found that while Myc bound to three sites, Hdac1 occupied only two of these three sites tested, while Gapdh bound to none (Fig. 7 F). These results suggest the strong possibility of *her4.1* being directly regulated through Myc–Hdac1 complex in the regenerating retina. We also quantified the *her4.1* mRNA levels in the sorted cells from the retina of *1016 tuba1a*:GFP transgenic fish treated with 10058-F4. Although there was significant decline in GFP^+^ MGPCs with 10058-F4 treatment (Fig. 3 G), we found a moderate increase in the *her4.1* mRNA levels in these GFP^+^ cells, and a substantial up-regulation was seen in GFP^−^ cells (Fig. 7 G). These observations suggest that the global increase in *her4.1* levels in the 10058-F4–treated retinae could be one of the reasons for lack of normal regeneration.

### Delta–Notch signaling restricts the zone of MGPCs by suppressing *lin28a* expression through Myc

**Figure 8.**
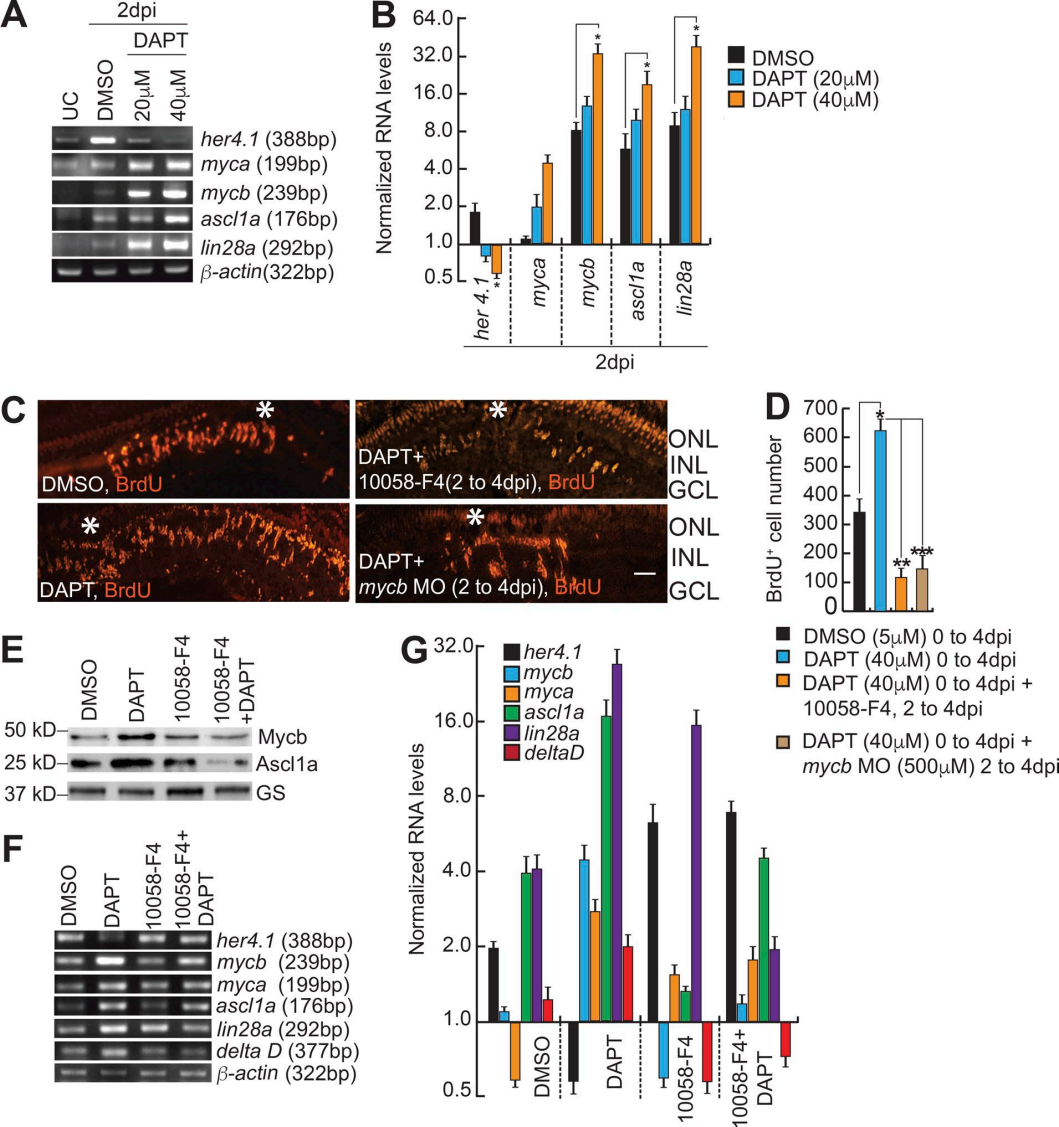
**Her4.1 restricts the zone of MGPCs by suppressing *lin28a* expression. (A and B)** RT-PCR (A) and qPCR (B) show decreased *her4.1* induction and increased regeneration-associated genes’ levels with DAPT treatment relative to DMSO control in 2-dpi retina. UC, uninjured control. *, P < 0.002. **(C and D)** IF microscopy shows that increased MGPCs seen in DAPT-treated (40 µM) retina is blocked both by 10058-F4 (10 µM) and *mycb*-targeting morpholino (500 µM; C) with ∼70% and ∼85% reduction in BrdU^+^ cells compared with DMSO control and DAPT-treated retina, respectively (D). *, P < 0.003; **, P < 0.0001; ***, P < 0.0002. Bars, 10 µm; white asterisks mark the injury sites (C). **(E–G)** Western analysis of Ascl1a and Mycb show reduction in protein levels, in 10058-F4 (10 µM) + DAPT- (40 µM) blocker regimen (E); also other regeneration-associated genes seen by RT-PCR (F) and qPCR (G) compared with DMSO, 10058-F4, or DAPT-treated retinae in 2 dpi. GS, glutamine synthetase. *n* = 3 biological replicates in all experiments. ONL, outer nuclear layer; INL, inner nuclear layer.

We next decided to investigate the mechanistic importance of Her4.1 up-regulation in Myc-Max–inhibited retina in detail. This could also enable us to find the reasons for reduced MGPCs in Mycb-compromised scenario. To decipher this, we first investigated the importance of Mycb and Lin28a in causing the increase in the number of MGPCs in post-injured retina with Delta–Notch signaling inhibition. The blockade of Delta–Notch signaling with DAPT treatment caused an enhancement of MGPCs, which was also accompanied by up-regulation of regeneration-associated genes like *myca*, *mycb*, *ascl1a*, and *lin28a* at 2 dpi (Fig. 8, A–C). Zebrafish embryos when treated with DAPT and injected with *lin28a:gfp-luciferase* reporter showed an increased *lin28a* promoter activity in a dose-dependent manner (Fig. S5 D).

**Figure 9.**
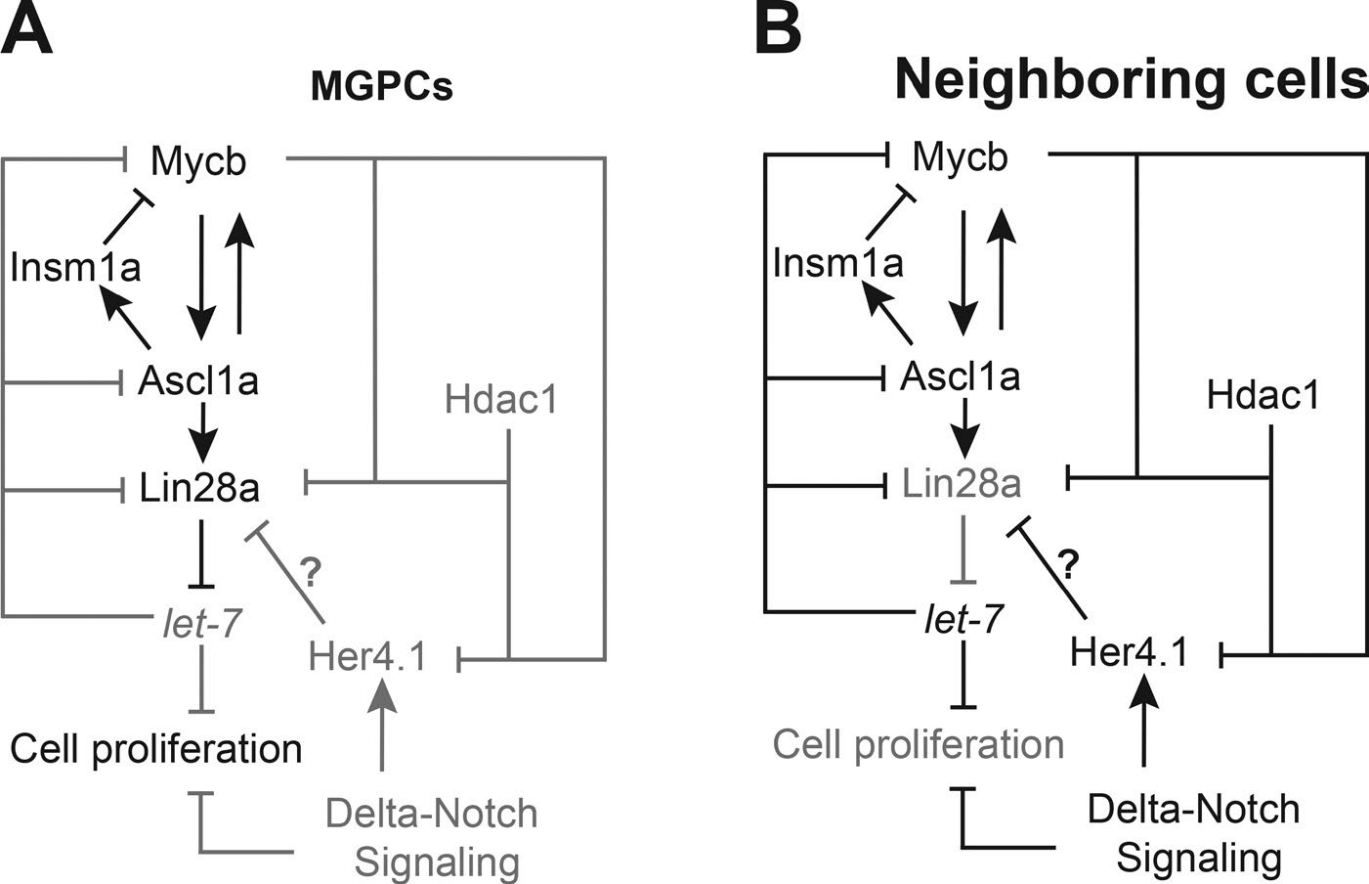
**The gene regulatory network mediated through Myc/Hdac/Ascl1a/Lin28a/Her4.1 in MGPCs and neighboring cells. (A and B)** The proposed model that depicts the mechanisms of genetic interaction of various regeneration-associated factors discussed in this study, shown separately in MGPCs (A) and neighboring cells (B) during retina regeneration. The interactions and molecules that are active are shown in black, and passive ones are in gray.

Interestingly, we found that the increased number of MGPCs, seen with DAPT treatment in 4-dpi retina could be abolished by Myc inhibition either by *mycb* MO or 10058-F4 exposures, in 0–4- and 2–4-dpi experimental regimes (Fig. 8, C and D; and Fig. S5, E–G). These observations suggested that the increase in the number of MGPCs seen in DAPT-treated retina was facilitated by normal Mycb-mediated gene regulations. We probed further the cause of reduction in cell proliferation through estimation of Ascl1a and Mycb protein levels in retina from double blocker experiments using DAPT and 10058-F4. We found that both Ascl1a and Mycb proteins were down-regulated in these retinae (Fig. 8 E). This prompted us to speculate that the reduction in MGPCs could be because of decline in one of the important regeneration-associated genes *lin28a* in the double blocker experiments as compared with DMSO control (Fig. 8, F and G). Furthermore, the reduced number of MGPCs also could be because of repression of the Notch ligand *deltaD* and increase in expression of *her4.1* (Fig. 8, F and G). Collectively, double blocker experiments suggested that Delta–Notch signaling is active in the vicinity of MGPCs to restrict the zone of proliferation, whereas Mycb-mediated signaling is active in MGPCs to increase the cell number. Since Myc is expressed in both of these cell types, its functional absence causes significant increase in Her4.1 (Fig. 8 G), reducing the number of MGPCs. Collectively, based on these results, we could assume that the Delta–Notch signaling and Mycb-induced regenerative mechanisms are independent in some MGPCs but dependent in others. The extensive gene regulatory network and their unifying proposed mechanisms are presented as a model (Fig. 9, A and B).

## Discussion

Proto-oncogene *myc*, a de facto transcription activator in various developmental programs (Yan et al., 2010) and pluripotency induction (Takahashi et al., 2014), is also known to cause gene-repression in some tumors (Herkert and Eilers, 2010). Moreover, Myc’s capability of epigenetic modifications makes it one of the unique transcription factors (Amente et al., 2011; Flaisher-Grinberg et al., 2012; Kozono et al., 2015; Matkar et al., 2015). Our studies suggest that Myc genes are unique, and especially, Mycb can act as an activator or a repressor regulating dedifferentiation of MG to MGPCs and their proliferation during retina regeneration. Furthermore, our studies unravel novel Mycb-mediated signaling mechanisms and gene induction paradigms, underlying MGPCs formation.

Quantitative analysis of coexpression of both *myca* and *mycb* along with PCNA^+^ cells in 4-dpi retina reveals that only 40–50% of MG-derived progenitors show *myca* and *mycb* expression. The juxtaposed *myca^+^/mycb^+^* cells should be the earlier MG cells that started dedifferentiating immediately after injury, which stayed restricted to injury site at 4 dpi, but some of *myca*^+^/*mycb*^+^ cells still may not enter the cell cycle. It is also important to note that up to 70% of *myca/mycb* expressing cells had PCNA expression. In other words, early progenitors would have *myc* expression, and late progenitors need not have it. Collectively, these results suggest that *myca/mycb* need not be present in all MGPCs, but a significant proportion of *myca*^+^*/mycb^+^* cells show proliferation. These observations also suggest the possibility of existence of a Myc-independent cell proliferation mechanism in the retina. This could also account for the lack of complete loss of MGPCs in Myc blocked retina, either by MO or by 10058-F4.

The disparity seen in the expression of *mycb* with *ascl1a* knockdown in early and late stages of regeneration may seem contradictory. This could be because of differential expression pattern of *mycb*, along with *ascl1a* and *insm1a*. The immediate early panretinal expression of *mycb* should be initiating the induction of *ascl1a*, which in turn induces *insm1a*, a repressor of *mycb*, *ascl1a*, and *insm1a* itself (Ramachandran et al., 2012). This negative feedback regulation seen within the first few hours of retinal regeneration abolishes the panretinal flash expression of *mycb*, *ascl1a*, and *insm1a.* During these early hours of regeneration, knockdown of *ascl1a* causes an increase in *mycb* expression because of lack of *insm1a* induction. In this period, through another pathway, Insm1a represses a panretinal Wnt inhibitor *dkk* that paves the way for initiation of Wnt signaling that could restrict the expression of *ascl1a* (Ramachandran et al., 2011) and *mycb* to the injury site at a later stage. However, at 4 dpi, the *insm1a* is not seen in every *ascl1a*^+^ MGPC; instead, its expression is restricted to a subset of cells that are about to exit cell cycle (Ramachandran et al., 2012). At this stage, upon *ascl1a* knockdown, *mycb* expression is down-regulated due to the lack of Insm1a in those *ascl1a*^+^ cells.

We uncovered specific roles of Myc during retina regeneration. First, Mycb activates *ascl1a* and regulates *lin28a* expression, which is essential to induce multiple regeneration-associated pathways (Ramachandran et al., 2010a; Zhang et al., 2016). Second, Mycb induces transcriptional repressors like *insm1a*, and inhibits *her4.1*, essential for fine-tuned expression of *ascl1a*, *lin28a*, and *mycb* itself, to the active zone of regeneration. Although canonical Wnt signaling–mediated up-regulation of Lin28a is shown in mammals (Yao et al., 2016), it may also induce Myc, which probably represses Lin28a either directly via Hdac1 recruitment or through Her4/Hes, as seen in zebrafish, reducing its regenerative potential. Moreover, in zebrafish retina, Ascl1a, which is a *wnt* inducer and β-catenin–regulated gene (Ramachandran et al., 2011), also contributes to the Lin28a level during retina regeneration (Ramachandran et al., 2010a). Seclusion of Hdac1 from BrdU^+^ MGPCs at 4 dpi, support the view that the role of Mycb in these cells would be as an activator of *lin28a* expression through Ascl1a. The opposite will be seen in Mycb^+^ and Hdac1^+^ cells at the vicinity of cell proliferation, wherein they collaborate to cause *lin28a* repression. These results affirm the dual roles of Mycb as a transcriptional activator and repressor on crucial genes like *lin28a*.

Closer investigation of regenerating retina with compromised Notch signaling reveals that Lin28a and Mycb cause enhanced number of MGPCs at injury site, which do not sustain in the absence of Mycb. Apart from its roles in mRNA splicing (Wilbert et al., 2012), and reprogramming cellular metabolism (Shyh-Chang et al., 2013), Lin28a also mediates down-regulation of *let-7* microRNA, essential for translation of several regeneration-associated genes (Ramachandran et al., 2010a), and Notch itself (Wang et al., 2010; Gökbuget et al., 2015), necessary for *her4.1* induction (Gemberling et al., 2013; Zhao et al., 2014a). Interestingly, our results show that Myc collaborates with Hdac1 to cause a decline in *her4.1* expression. The Her4.1 would probably suppress *lin28a* expression, causing a yin-yang relationship as part of restricting the zone of proliferation after focal injury. The results of double blocker experiments with DAPT and 10058-F4 in retina support this view. We find coexistence of increased *her4.1* and decreased *lin28a* levels when both Notch signaling and Myc were blocked simultaneously. This mechanism may also underlie the possible cause of lack of MGPC induction in Her/Hes overexpressed retina (Wan et al., 2012). Moreover, the decreased Lin28a levels could cause an increase in *let-7* microRNA in DAPT and 10058-F4–treated retina, which could bring down the protein levels of Myca, Mycb, and Ascl1a, as reported earlier (Ramachandran et al., 2010a), causing a reduction in the number of MGPCs.

Our data suggest that Myc plays important roles in different phases of retina regeneration (Fig. 9, A and B). First, it contributes in MG reprogramming to generate MGPCs through Ascl1a and Lin28a. Second, it restricts the zone of MGPCs through Her4.1–Lin28a axis. Finally, our studies unraveled important mechanisms by which Mycs and Hdacs mediate these effects through mutual signaling pathways, involving Ascl1a, Insm1a, Lin28a, and Her4.1, in retina regeneration. It is intriguing to speculate that GCL-specific rapid induction of *mycb* after optic nerve lesion also may significantly contribute to its regeneration in zebrafish. These studies suggest that Mycs and subsequent gene regulatory network are essential for retina regeneration, providing insights into signaling mechanisms that may help in understanding MG reprogramming in the injured mammalian retina, also with reference to damaged human retinae toward successful repair.

## Materials and methods

### Animals, fin cut, retinal injury, and drugs

Zebrafish were maintained at 26–28°C on a 14/10 h light/dark cycle. The *1016 tuba1a:gfp* transgenic fish used in this study have been previously described (Fausett and Goldman, 2006). Embryos for all assays were obtained by natural breeding. The Myc-Max inhibitor, 10058-F4, and Notch-signaling blocker, DAPT, were made to a stock of 1 mM in DMSO for various experiments (all drugs were from Sigma-Aldrich). Drugs were delivered either by dipping or injected into the eye using a Hamilton syringe with a 30-G needle. Retinal injury or optic nerve lesions were performed as described previously (Fausett and Goldman, 2006; Veldman et al., 2010). Fish were anaesthetized transiently in tricaine methane sulphonate, and the right eye was gently pulled from its socket and the retina stabbed four to eight times (once or twice in each quadrant) through the sclera with a 30-G needle inserted up to the length of the bevel. Optic nerve lesions were performed similarly, except that damage was not done to retina or blood vessel while cutting the optic nerve. Both retinal injury and optic nerve lesion were performed under a dissection scope (Stemi DV4; Zeiss). All experiments were done to a minimum of three times for consistency and SD.

### Primers and plasmid construction

All primers are listed in Table S1. The promoters of *mycb* and *her4.1* were amplified from zebrafish genomic DNA using primer pairs XhoI-*mycb* pro-F and BamHI-*mycb* pro-R (∼3 kb) or XhoI-*her4.1*pro-F and BamHI-*her4.1* pro-R (∼4 kb), respectively. The digested PCR amplicons were cloned into a pEL luciferase expression vector to create *mycb:gfp-luciferase* and *her4.1:gfp-luciferase* constructs. The *ascl1a:gfp-luciferase*, *lin28a:gfp-luciferase*, and *insm1a:gfp-luciferase* construct was described previously (Ramachandran et al., 2010a, 2012). The *lin28a* promoter site-directed mutagenesis was done as described previously (Ramachandran et al., 2010a). GFP was amplified from pEGFP-C1 plasmid with BamH1-EGFP-F and EcoR1-EGFP-R and cloned into pCS2^+^ vector.

Genes like *ascl1a*, *myca*, *mycb*, *insm1a*, and *lin28a* were cloned from cDNA amplified from zebrafish retina RNA at 4 dpi using primer pairs BamHI-*ascl1a* FL-F and XhoI-*ascl1a* FL-R (∼0.6 kb); BamHI-*myca*-F and XhoI-*myca*-R (∼1.2 kb); BamHI-*mycb*-F and XbaI-*mycb*-R (∼1.2 kb); BamHI-*insm1a*-F and XhoI-*insm1a*-R (∼1.1 kb); and BamHI-*lin28a* FL-F and XhoI-*lin28a* FL-R (∼0.6 kb). Post-digested PCR amplicons were cloned into their respective enzyme sites in pCS2^+^ plasmid to obtain *cmv:ascl1a*, *cmv:myca*, *cmv:mycb*, *cmv:insm1a*, and *cmv:lin28a.*

### Total RNA isolation, RT-PCR, and qPCR analysis

Total RNA was isolated from dark-adapted zebrafish retinae of control, injured, and drug-treated/MO-electroporated group using TRIzol (Invitrogen). Combination of oligo-dT and random hexamers were used to reverse transcribe 5 µg of RNA using Superscript II reverse transcription (Invitrogen) to generate cDNA. PCR reactions used Taq or Phusion (New England Biolabs) DNA polymerase and gene-specific primers (Table S1) with previously described cycling conditions (Ramachandran et al., 2010a). qPCR was performed in triplicate with KOD SYBR qPCR mix (QKD-201; Genetix) as per manufacturer’s recommendations on a real-time PCR detection system (Eppendorf Master Cycler RealPlex4). The relative expression of mRNAs in control and injured retinae was deciphered using the ΔΔCt method and normalized to ribosomal protein *l*-24 or *β*-*actin* mRNA levels.

### mRNA synthesis, embryo micro-injection, ChIP, and Co-IP assay

Various gene clones in pCS2^+^ plasmids having cDNA inserts were linearized, and capped mRNAs were synthesized using the mMESSAGE mMACHINE (Ambion) in vitro transcription system. For luciferase assay experiments, single-cell zebrafish embryos were injected with a total volume of ∼1 nl solution, containing 0.02 pg of *Renilla reniformis* luciferase mRNA (normalization), 5 pg of *promoter:gfp-luciferase* vector, and 0–6 pg of *ascl1a*, *insm1a*, or *mycb* mRNA. To assure consistency of results, a master mix was made for daily injections and ∼300 embryos were injected at single-cell stage. 24 h later, embryos were divided into three groups (∼70 embryos/group) and lysed for dual luciferase reporter assays (E1910; Promega).

ChIP assays to analyze endogenous Ascl1a or Mycb binding to various promoters in adult retina at 2 and 4 dpi were performed using ∼50 adult retinae after dark adaptation. Chromatin was isolated by sonication as described previously (Lindeman et al., 2009). The chromatin obtained after a brief fixing in 1% (vol/vol) formaldehyde for 10 min in room temperature and subsequent nuclear lysis were the starting material. The chromatin after sonication to make fragments of 500–800 bp in size was distributed into three equal aliquots; two were probed with an anti-zebrafish Myc and Ascl1a antibodies (described below), and the third served as a control. The antibody binding was done at 4°C with rotation. Using magnetic beads, the antibody bound chromatin were pulled down on magnetic rack. After washing, the chromatins were purified to obtain PCR-grade DNA using standard proteinase K, phenol chloroform extract before PCR analysis. Primers used for ChIP assays are described in Table S1. Co-IP was performed using the retinal lysate that were extracted using lysis buffer as per manufacturer’s recommendations and protocol reported elsewhere (Phizicky and Fields, 1995; Bonifacino et al., 2016). Co-IP was similar to ChIP in initial steps, except that the final eluted sample was run on an acrylamide gel, transferred onto a polyvinylidene fluoride membrane and probed with respective antibodies against proteins of the interaction complex obtained.

### Morpholino electroporation, mRNA transfection, and knockdown-rescue

Lissamine-tagged MOs (Gene Tools) of ∼0.5 µl (0.5–1.0 mM) were injected at the time of injury using a Hamilton syringe of 10-µl volume capacity. MO delivery to cells was accomplished by electroporation as previously described (Fausett et al., 2008). An ECM 830 Electro Square Porator (BTX) was used to electroporate the retina for MO delivery. BTX830 were adjusted to deliver five consecutive 50-ms pulses at 70 V with a 950-ms interval between pulses, using BTX electrodes of 0.5-cm diameter. The control and *ascl1a*-targeting MOs have been previously described (Ramachandran et al., 2012). Morpholinos targeting *myca, mycb and hdac1* are *myca* MO, 5′-AACTCGCACTCACCAGCATTTTGAC-3′; 2-*myca* MO, 5′-TTTAACGAATGCCGTTCCAGAATTG-3′; *mycb* MO, 5′-CCATACTTGAATTCAGCGGCATGGT-3′; 2-*mycb* MO, 5′-GAGTGCCGTAGCCGTGGTAAAAGCT-3′; *insm1a* MO, 5′-GCTTGACTAAAAATCCTCTGGGCAT-3′; and Ctl MO, 5′-CCTCTTACCTCAGTTACAATTTATA-3′.

Transfection mixture contained two solutions constituted in equal volumes: (1) 4–5 µg of mRNA mixed with HBSS and (2) lipofectamine messenger max reagent (LMRNA001; Invitrogen) mixed with HBSS. Both the solutions were allowed to stand at room temperature for 10 min and then mixed drop wise, followed by 30-min incubation at room temperature. The resultant solution was mixed with morpholino in equal proportion, and 0.5 µl of this mixture was used for injection in zebrafish retina, followed by electroporation as described earlier.

In vivo rescue experiments were designed for testing the specificity of *myca* and *mycb* MO antisense oligos. This was accomplished by transfection of zebrafish retina with gene-specific mRNA alongside the MO-targeting 5′ UTR region of concerned genes or control MO. For confirming the efficient mRNA transfection, GFP mRNA was also delivered by transfection in each experimental or control retina.

### BrdU/EdU labeling, retina tissue preparation for mRNA ISH, immunofluorescence (IF) microscopy, TUNEL assay, and Western blotting

BrdU labeling was performed by single i.p. injection of 20 µl of BrdU (20 mM) 3 h before euthanasia and retina dissection, unless mentioned specifically. Some animals required for long-term cell tracing experiments received more BrdU injections over multiple days. Fish were given higher dose of tricaine methane sulphonate, and eyes were dissected, lens removed, fixed in 4% PFA, and sectioned as described previously (Fausett and Goldman, 2006). mRNA ISH was performed on retina sections with fluorescein or digoxigenin-labeled complementary RNA probes (FL/DIG RNA labeling kit; Roche Diagnostics; Barthel and Raymond, 2000). Fluorescence ISH was performed according to the manufacturer’s directions (T20917, B40955, and B40953; Thermo Fisher Scientific). Sense probes were used in every ISH separately as control to assess the potential of background signal. IF microscopy protocols and antibodies were previously described (Ramachandran et al., 2012). IF microscopy was performed using rabbit polyclonal antibody against human ASCL1/MASH1 (ab74065; Abcam); rat monoclonal antibody against BrdU (ab6326; Abcam); mouse monoclonal antibody against human PCNA (sc-25280; Santa Cruz); rabbit polyclonal antibody against zebrafish Myca/b (Schreiber-Agus et al., 1993; AS-55477; Anaspec); rabbit polyclonal antibody against zebrafish Hdac1 (Harrison et al., 2011; Ab41407; Abcam); mouse polyclonal antibody against GFP (ab-38689; Abcam); rabbit polyclonal antibody against GFP (ab-6556; Abcam); and rabbit polyclonal antibody against mouse glutamine synthetase (Ramachandran et al., 2010b; ab93439; Abcam) at 1:500 dilution. Before BrdU IF microscopy, retinal sections were treated with 2 N HCl at 37°C for 20 min, equilibrated with 100 mM sodium borate (pH 8.5) for 10 min twice, and then processed using standard procedures (Senut et al., 2004).

Proliferating cells were labeled by intravitreal injection of 0.5 µl of 10 mM EdU solution dissolved in DMSO. A fresh injury was made near the cornea with a Hamilton Syringe of 10-µl capacity for intravitreal injection. Eyes were enucleated after 4 h, followed by cryoprotection as described elsewhere. EdU-labeled cells were detected by treating 8-µg retinal sections with Click-iT EdU Reaction cocktail (Click-iT TM EdU Alexa Fluor 647 Imaging kit; C10340; Thermo Fisher Scientific) prepared as per manufacturer’s instructions. In brief, after the fluorescence ISH (FISH) protocol, retinal sections were fixed with 4% PFA at room temperature for 20 min, followed by permeabilization with 1% BSA in PBS with Triton X-100 (PBST) at room temperature for 10 min and blocking with 3% BSA in PBST for half an hour. After blocking, 100 µl of Click-iT reaction cocktail was overlaid with glass coverslips for half an hour, followed by washing with 1% BSA in PBST. EdU-labeled cells were detected by confocal microscopy.

BrdU-labeled MGPC lineage-tracing experiments were done in retinal sections from single-eye sections of 8-µg thickness, distributed across five slides. Individual slide was first processed for IF-based detection of specific antigen or Mrna, and then BrdU or PCNA staining was performed as mentioned above using respective antibodies (Powell et al., 2012; Ramachandran et al., 2012). The total number of BrdU^+^ cells and the number of colabeled BrdU^+^ cells that also stained with a specific ISH probe and subsequent enzymatic reaction were quantified on each slide. TUNEL assay was performed on retinal sections using In Situ Cell Death Detection Fluorescein kit (11684795910; Roche) as per manufacturer recommended protocol. Western blotting was performed using whole retina tissue from four retinae per experimental sample, lysed in Laemmli buffer, size-fractioned in 12% acrylamide gel with SDS at denaturing conditions, before transferring onto Immun-Blot polyvinylidene fluoride membrane (162-0177; Biorad Catalogue), followed by probing with specific primary antibodies, and HRP-conjugated secondary for chemiluminescence assay using Clarity Western ECL (170-5061; Biorad Catalogue).

### Fluorescence and confocal microscopy and cell counting

After the staining experiments, the slides were examined with a Nikon Ni-E fluorescence microscope equipped with fluorescence optics and Nikon A1 confocal imaging system equipped with apochromat 60×/1 NA oil immersion objective lens. Imaging of bright field is done using Nikon DS-L3 camera attached onto the same microscope, as mentioned above. Cell counts were quantified by physically observing fluorescently labeled ISH, PCNA, or BrdU^+^ cells in retinal sections, visualized in the same microscope. We used 20× for low magnification and 40× or 60× oil objective with an NA set to 1 in almost all images. Images were from cryosections mounted on Super Frost Plus slides (Thermo Fisher Scientific), embedded with DABCO mounting medium in every retinal section discussed. The imaging was always done at room temperature. The confocal images were finally processed through deconvolution using the software NIS-Elements software and ImageJ. The final images were imported to Adobe Photoshop software (CC 2018) for conversion to 300 dpi. Every sections of the stained retina were mounted, observed, and analyzed, and at least three retinae from separate fish were used.

### Fluorescence-based cell sorting

RNA and Chromatin was obtained from FACS-purified MG and MG-derived progenitors at 4 dpi, as previously described (Ramachandran et al., 2011, 2012). In brief, uninjured and injured retinae were isolated from *1016 tuba1a:gfp* transgenic fish. GFP^+^ MGPCs from *1016 tuba1a:gfp* retinae at 4 dpi were isolated by treating retinae with hyaluronidase and trypsin and then sorted on a BD FACS Aria Fusion high speed cell sorter. Approximately 40 injured retinae from *1016 tuba1a:gfp* fish yielded 80,000 GFP^+^ and 170,000 GFP^−^ from DMSO-treated fish (20 retinae) and 40,000 GFP^+^ and 220,000 GFP^−^ (20 retinae) from 10058-F4–treated retinae.

### Statistical analysis

Observed data were plotted and analyzed using standard spreadsheet software (Microsoft Excel). All data, unless specified, represent mean with SD as error bar. Data distribution was assumed to be normal, but this was not formally tested. The statistical significance by comparisons of datasets was done using a two-tailed unpaired Student’s *t* test for all experiments. For all other comparisons, ANOVA was performed, and subsequently, a Bonferroni–Dunn post hoc *t* test was done using Stat View software.

### Online supplemental material

Fig. S1 shows the Myc and Max gene regulation during retina and optic nerve regeneration and development. Fig. S2 shows that knockdown of *myca* and *mycb*, separately and in combination, during regeneration decrease cell proliferation. Fig. S3 shows the rescue and expression dynamics of *myc* genes in retina and TUNEL assay with its blockade. Fig. S4 shows the regulation of regeneration-associated genes through Myc. Fig. S5 shows that Delta–Notch signaling and Myc show an interdependency during regeneration. Table S1 lists the DNA oligonucleotide primers used in this study.

## Supplementary Material

Supplemental Materials (PDF)
