# Supplementary material for: Dual regulation of lin28a by Myc is necessary during zebrafish retina regeneration
Source: J Cell Biol. 2019 Feb 4;218(2):489–507. doi: 10.1083/jcb.201802113 (PMC6363449; doi:10.1083/jcb.201802113)
Supplement: Supplemental Materials (PDF) [file JCB_201802113_sm.pdf]

Supplemental material

Mitra et al., https://doi.org/10.1083/jcb.201802113

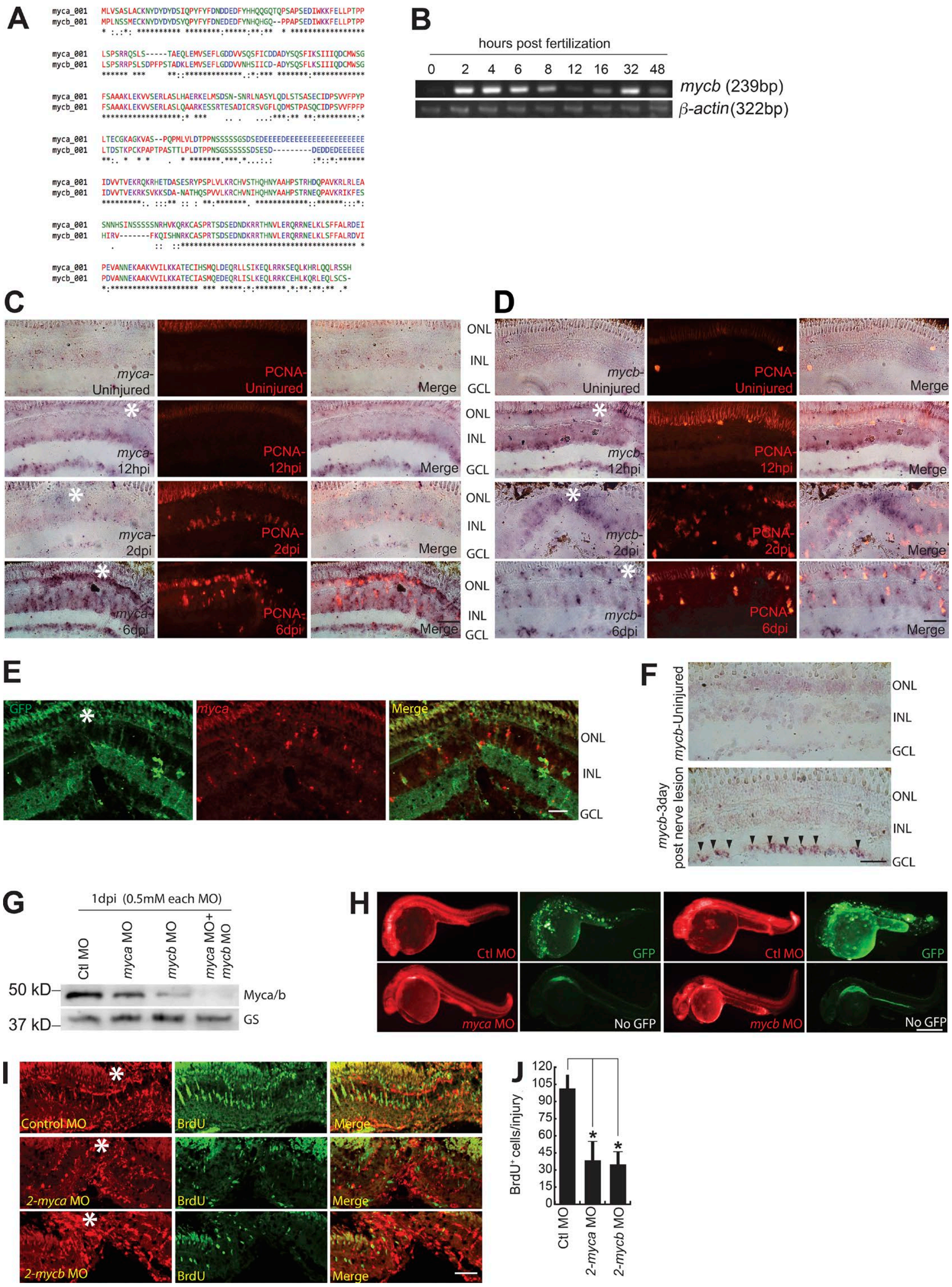

Figure S1. **Myc and Max regulation during retina, optic nerve regeneration and development. (A)** Amino acid sequence comparison between Myca and Mycb. **(B)** The *mycb* gene expression pattern during zebrafish embryo development. **(C and D)** ISH and IF microscopy of *myca* (C) and *mycb* (D), at various time points after retinal injury, show colabeling with PCNA in MGPCs and its neighboring cells. **(E)** Fluorescent ISH and IF microscopy show colabeling of endogenous *myca* with GFP$^+$ MGPCs in *1016 tuba1a:gfp* transgenic fish retina at 4 dpi. **(F)** ISH microscopy of *mycb* mRNA reveals GCL-specific induction on third day after optic nerve lesion, compared with uninjured retina. Black arrowheads indicate the GCL-specific *mycb* expression. **(G)** Morpholino-based antisense oligos against *myca* and *mycb* blocks Myc protein synthesis in zebrafish embryos; shown by Western blot analysis. GS, glutamine synthetase. **(H)** Fluorescence images of 24 h post-fertilization zebrafish embryos coinjected with control or gene-specific morpholinos and *gfp* mRNA, appended with MO-binding sequences, at single cell stage. MOs used were 0.5 mM each in all experiments. **(I and J)** Control (Ctl, 0.5 mM) or *myca*/*mycb*-targeting lissamine-labeled second MOs (0.5 mM) were electroporated into the retina of zebrafish at the time of retinal injury. Fish was given an intraperitoneal injection of BrdU 3 h before euthanasia at 4 dpi (I); quantification of the number of BrdU$^+$ cells at the injury site (J). The data are compared with control MO. *, $P < 0.0001$, $n = 4$ biological replicates. Bars: 10 µm (C–F and I) and 500 µm (H). $n = 6$ biological replicates. Error bars are SD. White asterisks mark the injury sites (C–E, H, and I).

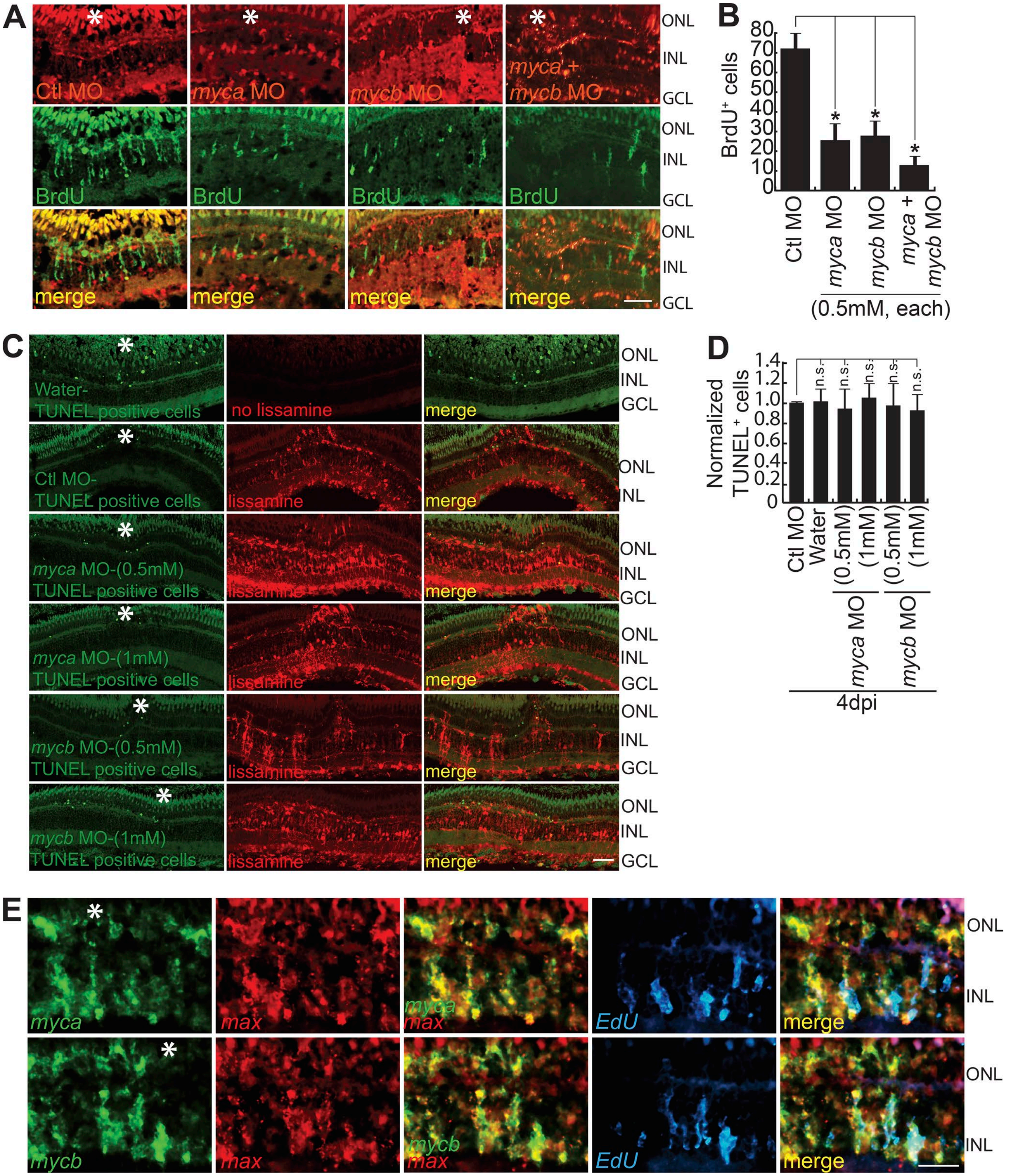

Figure S2. **Knockdown of *myca* and *mycb* separately and in combination during regeneration decrease cell proliferation. (A)** IF microscopy images of control (Ctl, 0.5 mM) or *myca*/*mycb*-targeting lissamine-labeled MOs (0.5 mM concentration each), electroporated into the retina of zebrafish at the time of retinal injury. Combined *myca* and *mycb* MOs together were of 0.5 mM concentration each. **(B)** Quantification of the number of BrdU+ cells at the injury site. The data are compared with control MO. *, P < 0.002; *n* = 4 biological replicates. **(C)** TUNEL assay shown by IF microscopy does not reveal an enhancement of apoptotic cells in *myca* or *mycb* MO-electroporated retinae, compared with normal or control MO-electroporated control retina at 4 dpi. **(D)** Quantification of TUNEL-positive cells from C. *n* = 6 biological replicates. **(E)** ISH and IF microscopy show that *max* gene expression show a colabeling with *myca* and *mycb* mRNA in EdU+ MGPCs and neighboring cells at 4 dpi. Error bars are SD. n.s., not significant. Bars, 10 µm; white asterisks mark the injury sites (A, C, and E).

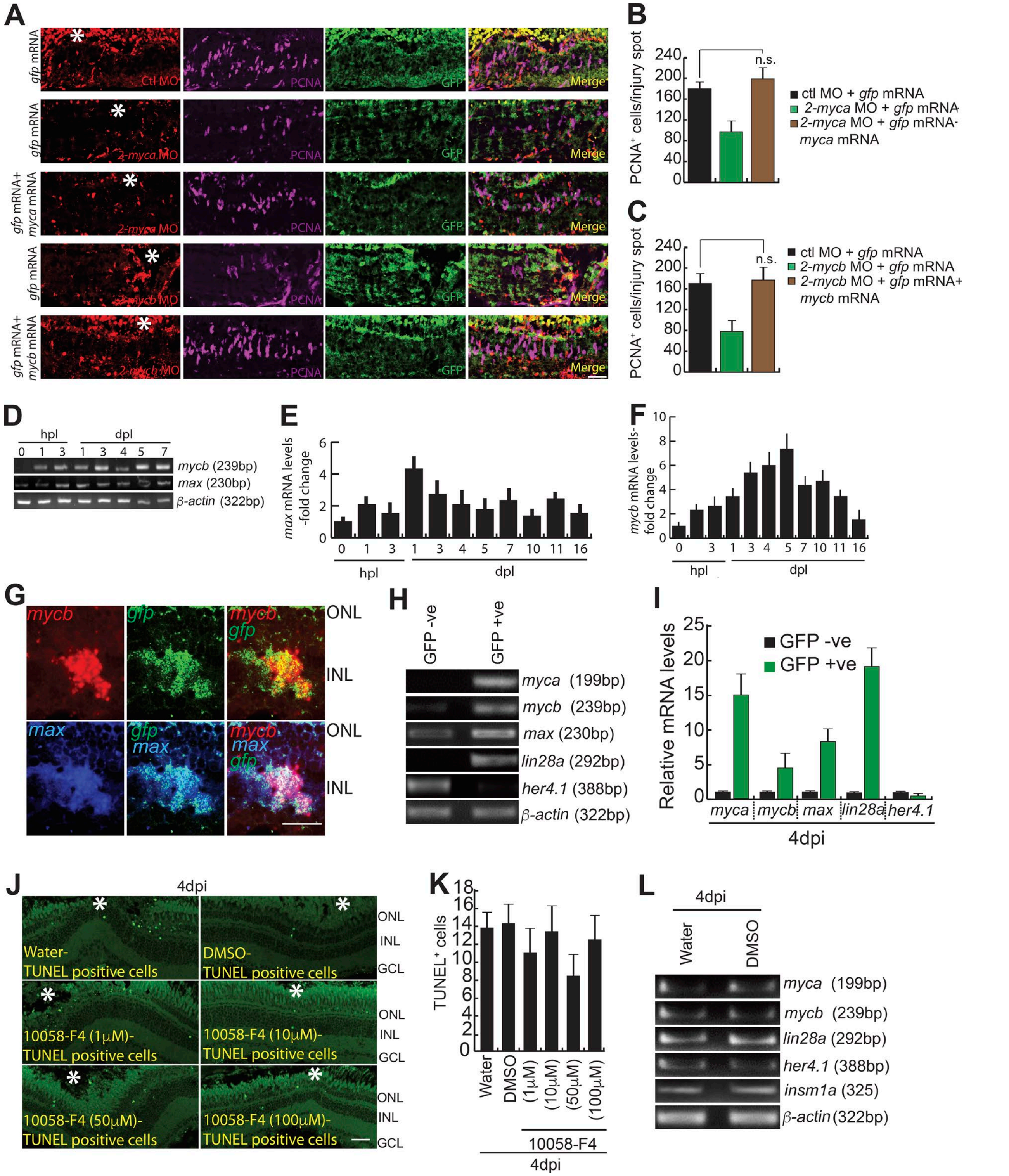

Figure S3. **Rescue and expression dynamics of *myc* genes in retina and TUNEL assay with its blockade. (A–C)** IF microscopy of retinal sections reveal the decrease in the number of PCNA+ cells by morpholino-mediated *myca* and *mycb* knockdown at 4 dpi during regeneration, which is rescued by respective mRNA transfected in situ (A) and quantified (B and C). n.s., not significant. **(D–F)** RT-PCR (D) and qPCR (E and F) analysis of *max* (D and E) and *mycb* (D and F) mRNA after optic nerve lesion, at various times after lesion reveals importance of possible Myc–Max interaction; *, $P < 0.03$. $n = 3$ biological replicates. hpl, h post lesion; dpl, d post lesion. **(G)** ISH microscopy image of *1016 tuba1a*:GFP transgenic retina that shows colabeling of *mycb* and *max* in GFP+ cells at 4 dpi. **(H and I)** RT-PCR (H) and qPCR (I) showing the expression levels of various regeneration-associated genes in GFP+ and GFP− cells from *1016 tuba1a*:GFP transgenic fish retina. **(J and K)** TUNEL assay shown by IF microscopy does not reveal an enhancement of apoptotic cells in 10058-F4–treated retina, compared with normal or DMSO-treated control retina at 4 dpi (J) and quantification of TUNEL-positive cells (K). **(L)** A gene expression comparison, by RT-PCR, of various regeneration-associated genes from retina of fish exposed to water or DMSO. Bars, 10 µm (A, G, and J). Error bars are SD. White asterisks mark the injury sites (A and J).

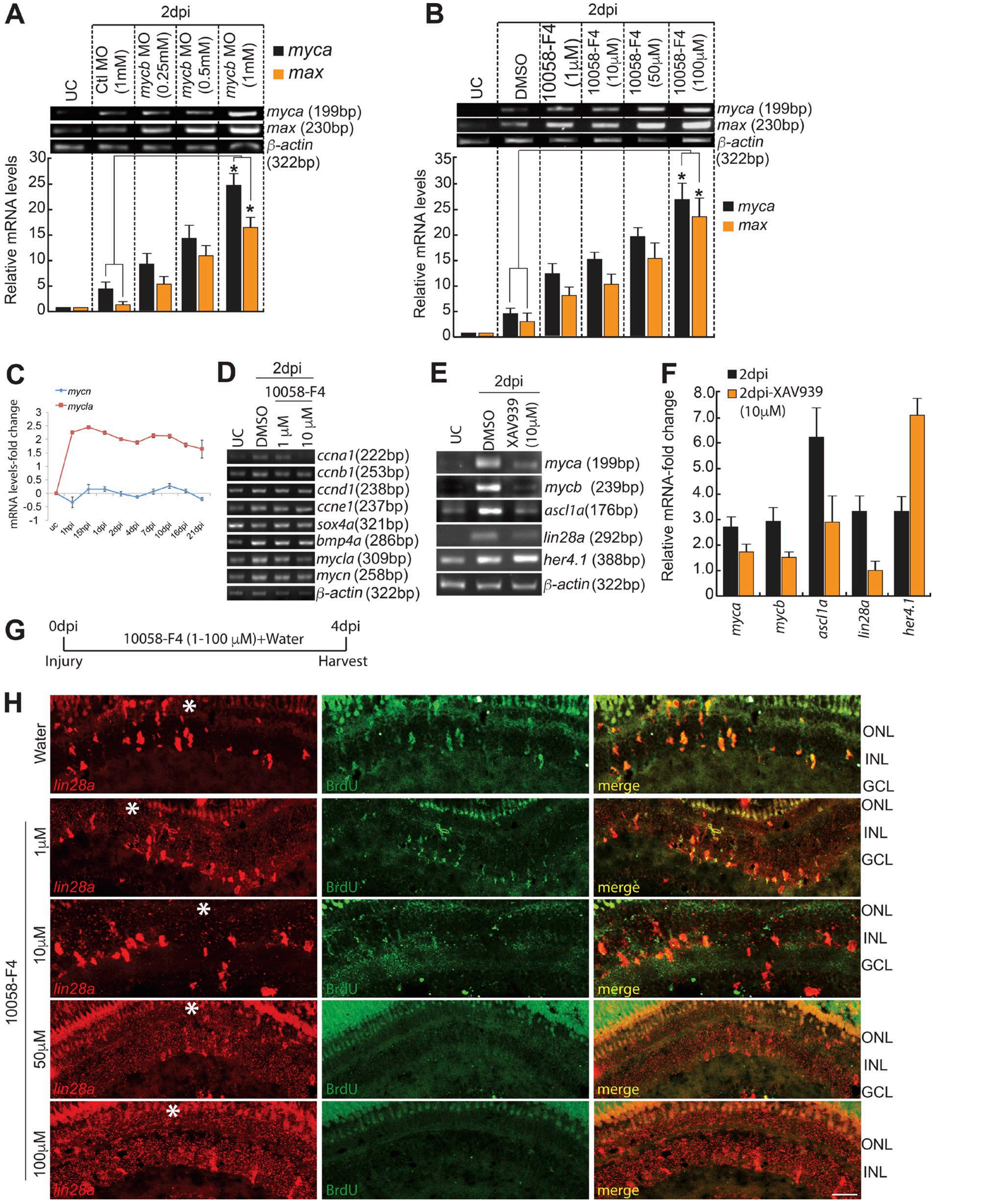

Figure S4. **Regulation of regeneration-associated genes through Myc. (A and B)** Mycb inhibition using antisense MO (A) or 10058-F4 (B) at 2 dpi causes an enhanced expression of *max* and *myca* (A and B), suggesting a feedback regulation, compared with control; *, P < 0.002 in A; *, P < 0.001 in B; *n* = 3 biological replicates. UC, uninjured control. **(C)** The qPCR assay of *mycn* and *mycla* genes at various times post retinal injury. **(D)** The RT-PCR assay of several Myc-influenced genes of retina with pharmacological inhibition of Myc–Max interaction using 10058-F4. **(E and F)** XAV939-mediated blockade of Wnt signaling, leading to inhibition of *myca* and *mycb*, represses several regeneration-associated genes and up-regulates repressor gene *her4.1*, which is confirmed by RT-PCR (E) and qPCR (F). **(G and H)** Experimental regimen of 10058-F4 treatment (G), to assay the *lin28a* mRNA levels by FISH in zebrafish retina at 4 dpi (H). Bars, 10 µm (H). Error bars are SD. White asterisks mark the injury sites (H).

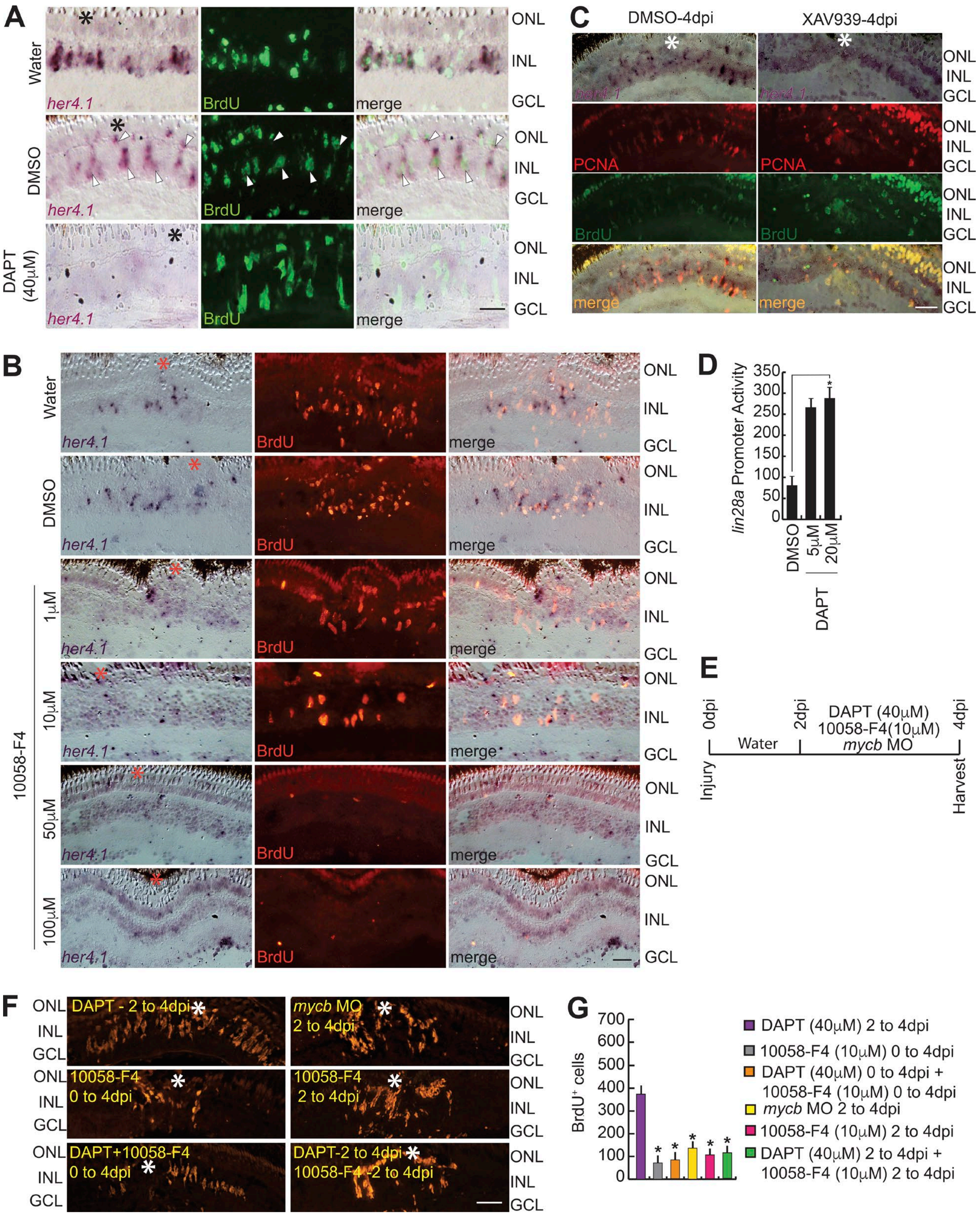

Figure S5. **Delta–Notch signaling and Myc show interdependency during regeneration. (A)** ISH and IF microscopy show that DAPT-mediated blockade of Notch signaling decreases *her4.1* induction compared with DMSO and water control in 4-dpi retina. Arrowheads indicate colabeled *her4.1*$^+$ and BrdU$^+$ cells. **(B)** ISH and IF microscopy shows dose-dependent increase in *her4.1* expression because of 10058-F4 treatment compared with water or DMSO-treated control in 4-dpi retina. **(C)** Expression of *her4.1* increases panretinally, seen by ISH microscopy in Wnt signaling blocked 4-dpi retina, with XAV939 (10 µM) compared with DMSO control. **(D)** Dose-dependent increase in promoter activity of *lin28a* with blockade of Notch signaling through DAPT treatment in zebrafish embryos, injected with *lin28a:gfp-luciferase*. *, P < 0.0002. **(E–G)** Late blockade of Notch signaling in injured retina with DAPT treatment, as shown in experimental time line (E), causes an increase in MGPC induction, which is blocked by Myc inhibition (F), revealed in cell quantification (G); *, P < 0.001. *n* = 6 biological replicates. Bars, 10 µm; black, white, and red asterisks mark the injury sites (A–C and F).

Table S1. **List of DNA oligonucleotide primers used in this study**

Full cDNA clone primers	Ensembl ID	Sequence 5′–3′
BamH1-Myca-F	ENSDARG00000045695	ATGCTCGAGGGATCCACCATGCTGGTGAGTGCGAGTTTGGC
Xho1-Myca-R		ATGGAGCTCTCGAGATGTGAACTCCGCAGCTGCTGAAGC
Mycb_F_BamH1	ENSDARG00000007241	ATGCTGAGGGATCCACCATGCCGCTGAATTCAAGTATGGAG
Mycb_R_Xho1		ATGGTAGCCTCGAGAGAACAGCTCAACTGCTCGAGTCTC
ascl1a FL Fwd	ENSDARG00000038386	ATGGACATCACCGCCAAGATGGAAATAAGCG
ascl1a FL Rev		TCAAAACCAGTTGGTGAAGTCCAGGAGCTC
lin28a FL BamHI F	ENSDARG00000016999	ATGCTGATGGATCCACCATGCCCCCGGCAAATCCGCATC
Lin28a FL XhoI R		ATGCTGATCTCGAGATCAGTGCTCTCTGGCAGTAAGGGAG
her4.1 FL Fwd	ENSDARG00000056732	GAAACTCTACTGACAAACAAGCTG
her4.1 FL Rev		GATGTTGTCCATCTTCGTTTAGTGC
BamH1-Insm-F	ENSDARG00000091756	ATGGTAATGGATCCACCATGCCCAGAGGATTTTTAGTCAAG
XhoI-Insm-R		ATGGTACTCGAGGCAGGCTGGACGCACCGGCATCTGAAG
**ChIP and Promoter primers**		
Ascl1a binding1 on Mycb pro Fwd		CACAGTTACTTTTATGGAAGGTAATGTG
Ascl1a binding1 on Mycb pro Rev		CGCTTTTCACTTTTGGTAATTGTTCC
Ascl1a binding2 on Mycb pro Fwd		GCAATATTTAGCTTGAGGCGAGGCA
Ascl1a binding2 on Mycb pro Rev		CACACATTCACACACACACTCATAAAC
Myc binding on Ascl1a pro Fwd		GAGAAGTTGTTTACTCAGTGGCAGGAG
Myc binding on Ascl1a pro Rev		CACTAAAGTTTTCTTTGGACTGCCC
Myc binding on Lin28a pro Fwd		GCTGTGGATCCTTTTCTCCTAAATGC
Myc binding on Lin28a pro Rev		CAGAACCAGGACCCTCTTCATATGATAATC
Myc binding on Her4.1 pro_1 Fwd		CAGCTTTTGGACATCTAAAAAGAGGTG
Myc binding on Her4.1 Pro_1 Rev		ATTTCTGAAAACAACATGTCATAAGGGC
Myc binding on her4.1 pro_2 Fwd		GCAGAGCTTAATTTGTAGCGTCCATAG
Myc binding on Her4.1 pro_2 Rev		AAAGTGACAGCAAATGCATTTATACCG
Myc binding on Her4.1 pro_3 Fwd		GCCCTCAGGAACAAGCTAAAATACCC
Myc binding on Her4.1 pro_3 Rev		GGCTGATACTGGGGTCATTTATTGAC
Xho-mycb pro-F		ATGCGTGTCTCGAGCGGGACAGTGCAGTCATGGGTCAG
Bam-mycb pro-R		ATGGCTGAGGATCCGGTGAGTGCCTGTGAAGAAGAAGGC
Xho ascl1aPro-F		CTCAGATCTCGAGAAAGATACACAAGCTGTCATTGGGGTGGTAC
Bam ascl1aPro-R		CCGCGGATCCTTCGCGGAGTCAAAAAAAGGAGTGAGTC
Xho-her4.1 pro-F		GGCTGAAGCTCGAGAACAAACAGACCATCAAAATGAAGTGTGAC
Bam-her4.1 pro-R		ATGCGTAGGGATCCTGCTGTGTGTCTTGTGTTCAGTTCTCCG
Xho-insm1a-Pro-F		CTCAGATCTCGAGTTGCCTTCAGAATAAATCACTAATGTCC
Bam-insm1a-Pro-R		CCGCGGGCCCGGATCCCTTCGCCAGCTGAAAGGCACTTCAGTCG
**qPCR Primers**	**Ensembl ID**	**Sequence 5′–3′**
myca RT Fwd	ENSDARG00000045695	AGCAGCAGTGGCAGCGATTCAGAAGATG
myca RT Rev		TGGAGACGTGACAGCGCTTCAAAACTAGG
mycb RT Fwd	ENSDARG00000007241	AGTAGTGACAGCGAATCCGATGACG
mycb RT Rev		ATGTGGCTCTCGAATTTAATCCGC
max RT Fwd	ENSDARG00000024844	ACGTAGGGACCACATCAAAGACAGC
max RT Rev		TGACTTTCTCCAGTGCCCGTACTTG
ascl1a RT Fwd	ENSDARG00000038386	ATCTCCCAAAACTACTCTAATGACATGAACTCTAT
ascl1a RT Rev		CAAGCGAGTGCTGATATTTTTAAGTTTCCTTTTAC
lin28a RT Fwd	ENSDARG00000016999	TAACGTGCGGATGGGCTTCGGATTTCTGTC

Table S1. **List of DNA oligonucleotide primers used in this study *(Continued)***

Full cDNA clone primers	Ensembl ID	Sequence 5′–3′
lin28a RT Rev		ATTGGGTCCTCCACAGTTGAAGCATCGATC
her4.1 RT Fwd	ENSDARG00000056732	GCTGATATCCTGGAGATGACG
her4.1 RT Rev		GACTGTGGGCTGGAGTGTGTT
insm1a RT Fwd	ENSDARG00000091756	CCAAGAAAGCCAAAGCCATGCGGAAGC
insm1a RT Rev		TTATTGCTTTCCGCGCTCTGCTTGGGTTTG
dld RT Fwd	ENSDARG00000020219	AAATGGAGGAAGTTGCACTGATC
dld RT Rev		AAGATCGAGACACTGAGCATCATTC
L24 Fwd	ENSDARG00000099104	CGACCCAGAGCAGCAAGG
L24 Rev		AGCACATCAGAGTTTAGC
b-actin Fwd	ENSDARG00000037746	GCAGAAGGAGATCACATCCCTGGC
b-actin Rev		CATTGCCGTCACCTTCACCGTTC
bmp4-RT-F	ENSDARG00000019995	GTGAGAGGATTCCATCATGAAGAGCAC
bmp4-RT-R		ACCTCAGCTCCCACACAAGAACCTCTG
mycla 201 RT Fwd	ENSDARG00000006003	CTGGACATTTTTGGGAATCTGGGTTC
mycla 201 RT Rev		ATCATCATCCTCATCGTCATCGGATG
mycn RT Fwd	ENSDARG00000006837	GAGACTCCGAGCGACTCTGATGATG
mycn RT Rev		GGCTGCTGCTGTCCTCTTTAAAATG
ccna1-RT-F	ENSDARG00000043236	GAAGTACAAGAGCTCGAAATATC
ccna1-RT-R		GATCGCTTTATAATCGTGCAC
ccnb1-RT-F	ENSDARG00000051923	GCCTTTCTAAGCATCTGGCTG
ccnb1-RT-R		CTTTTGCTCAACCCATGGCAG
ccnd1-RT-F	ENSDARG00000101637	GTCATCAGAAGTGACCCTGACTG
ccnd1-RT-R		CAAAGCCCATACCCATCAGAAAC
ccne1-RT-F	ENSDARG00000098622	CCTACTTGGAATGGCTGGGAAAG
ccne1-RT-R		CTTATGCCAATGCTTGTGAATGC
sox4a-RT-F	ENSDARG00000004588	AATCCAGCGACCCGCTGAGCTTGTAC
sox4a-RT-R		ACCTCAGCTCCCACACAAGAACCTCTG

https://doi.org/10.1083/jcb.201802113
